# Assessing the performance of weak and strong ion exchange solid-phase extraction and data mining tools to identify congeners and transformation products in municipal wastewaters by non-targeted analysis

**DOI:** 10.1007/s00216-026-06423-3

**Published:** 2026-03-25

**Authors:** Emmanuel Eysseric, Christian Gagnon, L. Mark Hewitt, Shirley Anne Smyth

**Affiliations:** 1 Aquatic Contaminants Research Division, Environment and Climate Change Canada, 105 McGill St., Montreal, QC Canada; 2 Aquatic Contaminants Research Division, Environment and Climate Change Canada, 867 Lakeshore Rd., Burlington, ON Canada; 3 Regulatory Operations, Policy, and Emerging Sciences Division, Environment and Climate Change Canada, 867 Lakeshore Rd., Burlington, ON Canada

**Keywords:** Non-targeted screening, SPE, Molecular networking, Compound Class Scoring, Polyoxyethylene congeners

## Abstract

Identifying contaminants in complex chemical mixtures requires powerful non-targeted analysis methods and tools, especially when it comes to detecting novel transformation products (TPs) and congeners from mixtures. In this study, we provided a comparison of weak versus strong ion exchange solid-phase extraction (SPE) for non-targeted accurate mass profiling of contaminants from wastewater samples and evaluated two data mining tools—Molecular networking and Compound Class Scoring (CCS)—for identification and annotation of transformation products and congeners across multiple municipal wastewater treatment plants. Weak ion exchange resins paired with hydrophilic-lipophilic balance resins showed superior performance in terms of higher average recoveries of spiked internal standards and provided enhanced sensitivity for key contaminant classes including surfactants, pharmaceuticals, and recreational drugs. This was reflected by both greater peak areas and the number of compound identifications. Using these techniques, >800 contaminants were identified at various levels of confidence. Consumer product additives and pharmaceuticals accounted for most of the contaminants in terms of number of identified compounds and abundance. Over 50 TPs of pharmaceuticals and multiple subfamilies of consumer product additive congeners were identified with molecular networking. Polyoxyethylene congeners—namely alkyphenol ethoxylates and linear alkyl ethoxylates—were found in high quantities using CCS, which uses both data-dependent and data-independent acquisition tandem mass spectrometry (MS^2^) data. The tool showed strong proficiency for large-scale identification of polyoxyethylene congeners, as over 2000 unique compounds were detected. Despite the abundant MS^2^ information, only 9% of these compounds were identified, which underscores the challenge posed by complex industrial mixtures.

**Graphical abstract:**

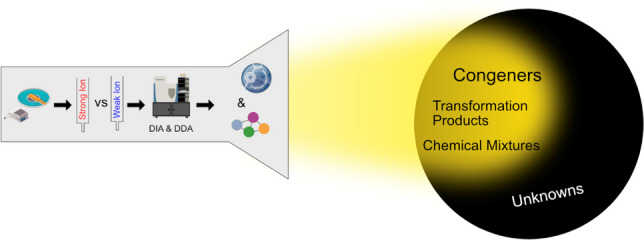

**Supplementary Information:**

The online version contains supplementary material available at 10.1007/s00216-026-06423-3.

## Introduction

Globally, there are hundreds of thousands of substances registered for domestic production and commercialization that could be released in the environment, not including potential transformation products (TPs) [1]. A major way of entry for a number of contaminants in water bodies is through urban wastewater effluents. A broad range of contaminant molecular structures can be found in wastewater treatment plant (WWTP) effluents, ranging from small pharmaceuticals to large industrial oligomers [2–4]. Over 350 L per person per day of treated and untreated wastewaters was released in 2020 in Canada with chemical oxygen demands ranging between 18 and 174 mg/L for treated effluents [5]. This puts in perspective the sheer mass of organic loadings from WWTPs to aquatic environments, with most of it remaining uncharacterized [5].

The challenge in the identification of contaminants is compounded by the matrix effects of the effluents [4]. This analytical challenge to qualify and quantify the organic contaminants and their TPs is immense with numerous difficulties such as commercially unavailable reference standards, unknown TPs, and the sheer number of compounds present. Indeed, tens to hundreds of industrial homologues such as surfactants, lubricants, and polyfluoroalkylated substances (PFAS) have been found in WWTP effluents as well as urban and industrial runoff [3, 6, 7]. As traditional targeted methods cannot sustain the growing analytical burden required to document this contamination, new non-targeted analysis (NTA) has become a useful tool in characterization techniques [2–4, 8]. NTA of WWTP effluents and runoff in the literature either takes the form of (i) suspect screening in which compounds from specific classes are searched, or (ii) non-targeted screening which attempts to identify all the contaminants detected [3, 4, 8–17] Thus, it is essential that extraction methods in non-targeted screenings assays achieve maximal and broad recoveries [18].

Solid-phase extraction (SPE) is the most commonly used technique for the preparation of aqueous samples [18]. There is however a high variance regarding the choice of sorbents used; while neutral sorbents such as Strata-X or Oasis HLB are widely used, sorbents containing ion-exchanging moieties such as anionic and cationic exchange sorbents are applied less frequently [2, 3, 6–17]. Moreover, while both weak and strong ion exchange cartridges are used in non-targeted methods, weak ion exchange sorbents seem to be favoured in environmental analysis whereas strong ion exchange sorbents see more use in biological matrices [2, 10, 11, 16, 19, 20]. There is, to our knowledge, no information available on the comparative performance of weak vs strong ion exchange sorbents for non-targeted analysis of wastewater samples, especially when it comes to “blind spots”—compounds not amenable, or weakly amenable to ion exchange. For example, low recoveries may occur for compounds with low pKa values (e.g., perfluorooctanoic acid (PFOS) or alkylbenzenesulfonates) with strong anion exchange sorbents while organic acid transformation products of pharmaceutical compounds might be less retained by weak anion exchange sorbents. An issue arises as these aforementioned compounds are all highly likely to be found in wastewater effluents [2, 3]. Although this might be less of an issue for suspect screening studies of specific classes of contaminants, where the scope of the employed methods is limited and often guided by the classes of contaminants targeted, the best compromise ought to be determined for non-targeted screening to preserve its purpose of identifying unknowns from multiple classes.

Non-targeted screening (NTS) methods use high-resolution tandem mass spectrometers in data-dependent acquisition (DDA) to automatically generate high-resolution MS^2^ (HRMS^2^) spectra that are then matched against spectra from HRMS^2^ databases for identification purposes. These HRMS^2^ databases are constrained by the number of compounds they contain, especially when it comes to transformation products and congeners of industrial compounds. As such, with a highly variable and the increasingly dynamic mixture of contaminants in WW, there is a need for suitable approaches. One such tool is molecular networking, which uses MS^2^ spectra generated by DDA and groups precursor ions in networks based on their MS^2^ spectra similarity [21–23]. Molecular networking is highly complementary with HRMS^2^ databases as they can assist in the identification of TPs and industrial homologues [6, 7, 24, 25].

Nevertheless, with more complex matrices such as WW influents and effluents, DDA experiments may fail to gather MS^2^ spectra of potentially important contaminants if there are sufficiently high coeluting substances. One workaround to counter this phenomenon in highly complex mixtures is data-independent acquisition (DIA), where all precursor ions are sequentially selected for an MS^2^ experiment. DIA can provide additional complementary information in the form of MS^2^ spectra with more product ions for a higher number of compounds with more reproducibility than DDA, at the expense of more complex MS^2^ spectra [18]. While DIA has been used in surface water analysis, there are several shortcomings in the analysis of small and structure-diverse molecules in complex matrices such as WW effluents [14, 26]. For example, the attribution of the proper product ions to the right precursor with multiple coeluting species in a given scan and the limited frequency of acquisition of some high-resolution instruments are known issues [18]. It is then no surprise that most software solutions geared toward the identification of small molecules—mzCloud and mzMine for example—offer limited integration of DIA data in their identification workflows. However, Compound Discoverer software from Thermo® uses DDA and DIA data with the Compound Class scoring tool, which allows ranking and organizing features based on the presence of common fragment ions in their MS^2^ spectra. Compound class scoring has been used in suspect screening studies to search for substances of abuse and PFAS in WW effluents, but not in non-targeted screening studies nor in combination with DIA [27–30]. While the targeted aspect of Compound Class Scoring makes it seemingly more suited for suspect screening analysis with DDA, this tool could potentially also benefit non-targeted screening in order to assist in the identification of particularly large classes of compounds like PFAS, oligomers and linear alkyl sulfonates.

This study aims to address gaps in extraction and data workflows described above regarding ion exchange sorbents for SPE of wastewaters and comparative assessments of molecular networking and compound class scoring for the identification of congeners and TPs. The first objective of this study was to assess which ion exchange sorbent provides the optimal broad recovery for a NTS method in a complex matrix of wastewater samples. To this end, the performance of strong ion exchange and weak ion exchange sorbents paired with hydrophilic-lipophilic balance neutral sorbents was evaluated in WW effluents and spiked WW effluents using multiple performance indicators. The second goal was to evaluate the efficiency of two data mining tools, molecular networking and compound class scoring, using DDA and DDA paired with DIA data respectively, to enable the identification of industrial congeners as well as TPs in influents and effluents from WWTPs of variable size and treatment types. A final objective was to assess several of the analytical challenges in non-targeted screening of wastewater samples regarding identification of unknowns.

## Materials and methods

### Sampling

Samples were collected at six WWTPs of varying size, inputs and treatments (Table S1). Three refrigerated 24-h equal-volume composites were collected for influents and effluents over three contiguous days at plants QE, HG, and SK, while grab samples were collected at PV and NW, totaling six samples per WWTP. At plant BF, only composite effluents were collected. These sampling techniques have been previously published [31]. Samples were stored in 250 mL amber glass bottles and refrigerated at around 4 °C until reception at the laboratory, whereupon a small volume was withdrawn to prevent bottle breakage and storage was at −20°C until extraction. No preservatives were added.

### Extraction

A survey of extraction methods used in non-targeted analysis studies of wastewaters was conducted in order to assess the main types of SPE sorbents used and is shown in Table S2.

#### Solid-phase extraction optimization

Prior to extraction, samples from the QE WWTP were thawed to room temperature and filtered with 0.22 µm PVDF Stericup® Quick Release Vacuum Driven Filters from Sigma Millipore (Saint-Louis, MO), and the pH was buffered to 7. To the 100 mL filtrate, 50 µL of internal standards (Table S4) solution (concentration of 10 mg/L in methanol) was added. The samples were then extracted in two sets of three SPE cartridges in series. All cartridges had been conditioned and equilibrated beforehand with 6 mL methanol (Optima LC–MS, Fisher Scientific) and 6 mL milli-Q water respectively. The first set, the Strong Ion Exchange (SIE), was composed of an Oasis MCX (150 mg 30 µm), an Oasis MAX (150 mg 30 µm), and an Oasis HLB (200 mg 30 µm) cartridge, while the Weak Ion Exchange (WIE) set was composed of an Oasis WCX (150 mg 30 µm), an Oasis WAX (150 mg 30 µm), and an Oasis HLB cartridge (150 mg 30 µm), in those orders. Samples were loaded by gravity, unless a vacuum was required, with the flow rate of the terminal HLB cartridge set at 1 drop/s maximum. A picture of the WIE set up is shown in Figure S1. A wash step was done using 6 mL milli-Q H_2_O. A mild wash step was chosen in order to retain as many polar compounds as possible as used in previous studies, though at the potential expense of adverse matrix effects [18]. Elution for the HLB was done with 2 × 3 mL acetonitrile 2% formic acid. For the MCX and WAX cartridges, elution was done with 5% ammonium hydroxide in methanol and 2% formic acid with acetonitrile for the MAX and WCX cartridges. Acetonitrile was used as the elution solvent for the acidic eluent in order to limit Fisher esterification in the elution solution [32]. Samples were combined then dried under nitrogen flow and reconstituted in 500 µL of 9:1 H_2_O (Optima LC–MS) and methanol (Optima LC–MS). The method with the better performance was selected for the following section. The criteria for determining which procedure had better performance were higher recoveries of spiked internal standards, enhanced sensitivity for key contaminant classes, higher number of identified compounds with Compound Discoverer databases, and higher number of transformation products and congeners in molecular networks.

#### Non-targeted screening of effluents from multiple treatments

Samples from all WWTPs were extracted for NTA using the optimized method with the exception of a different solution (Table S5 with a concentration of 10 mg/L in methanol) and 500 mg cation and anion cartridges (WCX and WAX; 500 mg, 60 µm).

### Wastewater treatment plants

Details about WWTPs investigated in this study are shown in Table S1. Plants were selected to represent a broad range of average flow (400 to 161,460 m^3^ day^−1^), type of input (range), system hydraulic retention time (45 min to up to 6 months), phosphorus removal capacity (range) or lack thereof.

### Liquid chromatography

Chromatographic separations were achieved using a 2.1 × 150 mm XSelect HSS T3 2.5 µm (Waters, Milford, MA). Mobile phase A was H_2_O with 0.1% (v/v) formic acid and mobile phase B was 1:1 (v/v) methanol:acetonitrile with 0.1% (v/v) formic acid for the positive mode. For the negative mode, mobile phase A was H_2_O with 5 mM ammonium acetate and 0.1% ammonium hydroxide while mobile phase B was 1:1 (v/v) methanol:acetonitrile with 5 mM ammonium acetate and 0.1% ammonium hydroxide. Elution conditions were the same for positive and negative electrospray ionization (Table S3).

### Mass spectrometry

A Q-Exactive plus quadrupole-Orbitrap mass spectrometer was used for non-targeted analysis (Thermo-Fisher, Waltham, MA). Acquisitions were carried out in both positive and negative ionization modes. Full scan mode was used for semi-quantitation as the faster acquisition rate and higher resolution allowed for an optimal number of points per peak as well as a better separation of isobars. DDA settings were selected based on previous studies on Q-Exactive platform parameters for optimal library identification and molecular networking formation by Stincone et al. [33]. DIA was used to generate additional MS^2^ information, notably for the ions with a lower intensity that would not have been selected for data-dependent acquisition MS^2^. Further details on all ionization and data acquisition settings are provided in SI.

### Quality control

#### Internal standards

Stable isotope labeled internal standards of varying polarity were used to assess extraction efficiencies for the evaluation of the extraction methods (Table S4) and for the non-targeted screening of the 6 WWTPs to assess extraction efficiencies, instrument performance and for data normalization (Table S5).

#### Nontargeted Analysis Study Reporting Tool

The *Nontargeted Analysis Study Reporting Tool* (Table S6) was used to design the quality control steps as well as the overall scope of the method, acquisition, data analysis and reporting [34]. This tool offers a guideline for study design, data acquisition, data analysis, visualization and results presentation and QA/QC metrics. The objective was to obtain a score of 3, when applicable, in all the subsubsections corresponding to best practices in non-targeted analysis.

### Data analysis, software, treatment, and visualization

Multiple workflows were used for data analysis and treatment, as well as software for visualization. Briefly, full scan files were used for peak area comparisons while data-dependent and data-independent acquisition files were used for compound annotation. Data-dependent acquisition files were used for library search, molecular networking, and compound class scoring while data-independent acquisition files were used for library search and compound class scoring. Data-independent acquisition files were unfortunately not compatible with molecular networking using mzMine. The open access software MS-Dial can be used for deconvolution of DIA MS spectra followed by molecular networking; this should be further considered in future studies [35, 36]. Details on file conversion, networks parameters in GNPS and Cytoscape, workflow nodes in Compound Discoverer are provided in the SI and Fig. S2.

## Results and discussion

As our first objective of this study, we assessed ion exchange sample extractions suitability for NTA. The weak ion exchange method showed a higher performance based on the selected criteria and was then applied on WW samples from different treatment types with the results of over 800 compounds tentatively identified. Our second objective was to then evaluate molecular networking and compound class scoring tools for MS^2^ identification, which allowed identification of transformation products and congeners, where, in most instances, MS^2^ databases could not furnish an annotation. The limitations of both tools in the context of this study were explored as our final objective.

### Solid-phase extraction optimization

**Fig. 1:**
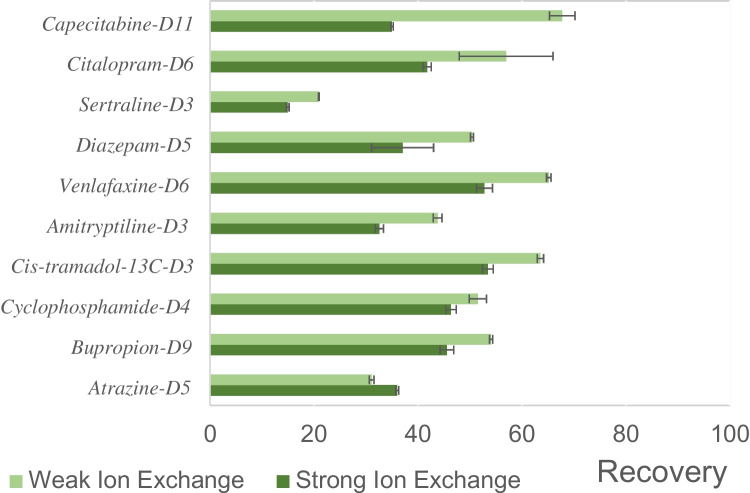
Recoveries for the spiked stable isotopes internal standards. Error bars are standard deviation (n = 7)

The comparative assessment of ion exchange resin recoveries from effluent samples was the first performance criterion. Weak ion exchange resins (WIE) in series with the hydrophilic-lipophilic balance (HLB) resin outperformed the strong ion exchange (SIE) in series with the HLB resin, as recoveries were 11% higher on average with 50.5% and 39.5% for the WIE and SIE, respectively (Fig. 1). Indeed, only 2 of the 10 internal standards spiked in the samples had recovery > 50% with the SIE cartridges whereas 7 out of 10 were > 50% with the WIE cartridges. Low recoveries were observed with sertraline-D3, atrazine and amitriptyline with both ion exchange resins. These results are somewhat counterintuitive, especially with the SIE resins, considering the physicochemical properties of the compounds (Table S4). While it could seem that secondary and tertiary amines are less retained, significantly higher recoveries were measured for venlafaxine (secondary amine), bupropion and tramadol (tertiary amine) which have similar pKa. The largest difference in recoveries between resins was for capecitabine, with over 30% higher recovery for the weak ion exchange. This is consistent with the expected performances of both sorbents, because the lower pKa of the compound (1.9) is below the effective range of the medium anion exchange cartridge used in the SIE workflow [37]. Except for sertraline, atrazine and amitriptyline, all spiked internal standards had over 50% recovery, which is satisfactory for non-targeted screening methods [18].

**Fig. 2:**
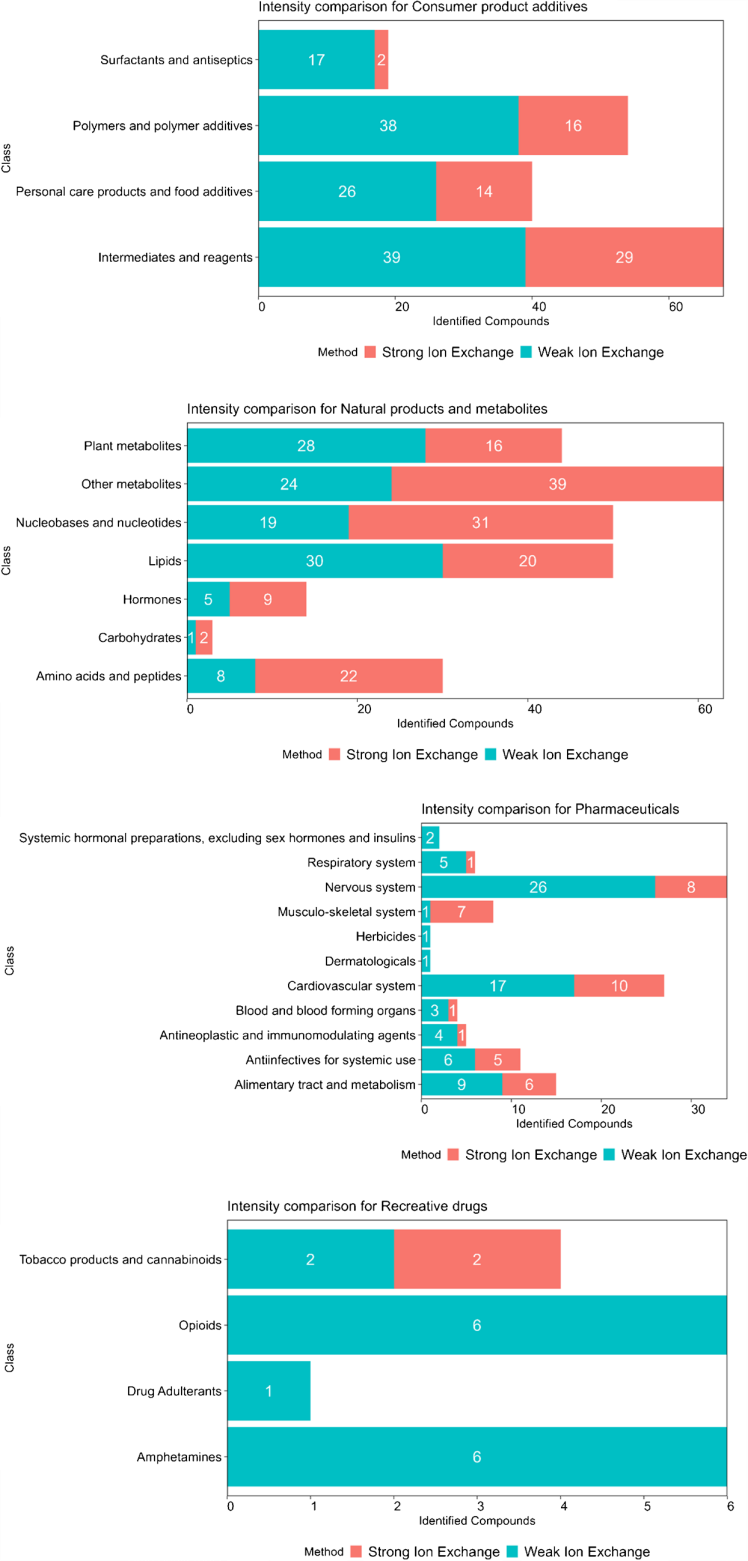
Comparison of the signal intensity for compounds identified with mzCloud and mzVault nodes per superclass and class with both extraction methods. For example, of the 19 surfactants and antiseptics compounds identified, the signal for 17 of them was higher with the Weak Ion exchange extraction method versus 2 with the Strong Ion exchange extraction method

A non-targeted screening workflow was then applied to the samples extracted with both methods. Features that exceeded a matching score of 70 with Compound Discoverer using mzCloud and mzVault—the integrated online and offline MS^2^ library matching tools on Compound Discoverer—were filtered, in order to maximize reproducibility, with improbable or low-quality annotations subsequently withdrawn. A matching score represented how well the spectrum of the unknown ion overlaps with the spectrum of the database reference standard. As shown in Fig. 2 and Table S10, the WIE outperformed the SIE for consumer product additives (especially surfactants and antiseptics), pharmaceuticals and recreative drugs where 66%, 66%, and 88% of features were more intense, respectively. For these superclasses, peak areas were over 1.5 times higher 23%, 11%, and 35%, respectively (Table S10). The SIE method appeared to excel for natural products and metabolites, where 55% of annotated compounds were more intense using that method and 35% were over 1.5 times more intense (Table S10). These results are consistent with the preferred use of SIE sorbents with biological samples or in metabolomic studies.

These results highlight the compromises required with extraction across broad compound classes in such complex mixtures. In the context of non-targeted screening of wastewater samples without a particular focus on metabolites, Weak Ion Exchange sorbents show higher performance for consumer product additives, pharmaceuticals, and recreative drugs.

Following this initial work of comparing matrix recoveries with labelled reference standards, we then investigated the performance of both extraction methods for specific contaminant classes or individual substances. The peak areas of a family of cardiovascular system drugs—angiotensin II receptor blockers—were on average 27% higher with WIE sorbent while another family of cardiovascular system drugs, beta-blockers, were 3% higher with the SIE. The peak areas of alkyl and aryl sulfonates, used as surfactants, were on average 45% with the WIE method as well as for the PFAS, which had peak areas 34% higher with the WIE method. Based on these results, WIE was superior across the classes examined.

The performance of both methods was then further evaluated, as a third step, with the use of molecular networking. Compounds previously tentatively identified with Compound Discoverer were examined in molecular networks in search of transformation products and congeners that would be structurally linked. In so doing, this performance criterion would reflect the ability of both extraction methods to retain multiple compounds from a family of congeners or group of TPs. As shown in Table 1, 59 and 52 compounds were found in molecular networks with WIE and SIE methods respectively, which correspond to 45 and 38 tentatively identified congeners and transformation products when excluding those already identified with Compound Discoverer’s mzCloud and mzVault nodes. All individual molecular networks are shown in SI. When separating per superclass (that groups multiple classes), WIE was generally similar to, though slightly better than, SIE with 21 vs 17 pharmaceuticals and 35 vs 32 consumer product additives, respectively.

**Table 1:** Number of compounds found by molecular networks in the QE-EFF samples with the Weak and Strong Ion exchange solid-phase extraction methods. Molecular networks were generated from merged features for each extraction method where n = 3

Network	Weak ion exchange	Strong ion exchange
Diltiazem and transformation products	1	4
Angiotensin II receptor blockers and transformation products	7	4
Atorvastatin and transformation products	4	3
Beta-blockers and transformation products	9	6
Cocaine and transformation products	3	3
Tris(2-butoxyethyl)phosphate and transformation products	7	6
Alkylamidopropyl betaines congeners	5	4
Alkyl diethanolamines congeners	5	6
Nonylphenol ethoxylates congeners	13	13
Nonylphenol ethoxy acids congeners	5	3

In conclusion, WIE extraction slightly outperformed SIE extraction, with higher average matrix recoveries of spiked consumer product additives, pharmaceuticals, and recreational drugs. This was reflected in both higher peak areas and a greater number of identified compounds and transformation products within these groups. Conversely, SIE cartridges proved more effective for extracting natural products. Considering that consumer product additives and pharmaceuticals arguably form the two main superclasses of contaminants of concern in municipal wastewaters, the consistently superior results in both categories, with higher peak areas and a greater number of identified transformation products and congeners with the WIE method, unequivocally point toward this extraction type for further similar studies.

### Non-targeted screening of multiple wastewater treatment plants influents and effluents with weak ion exchange cartridges

**Fig. 3:**
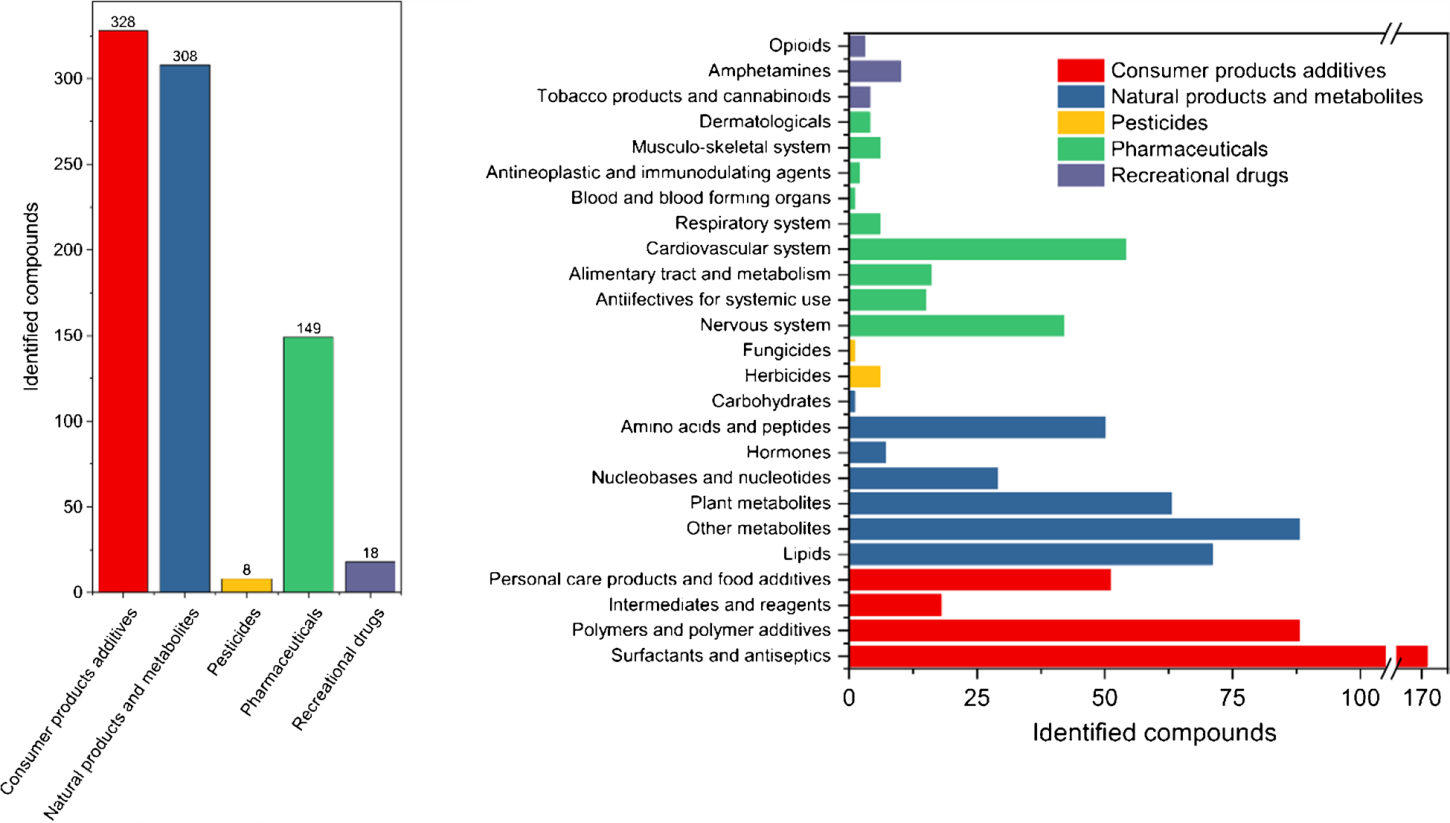
Tentatively identified compounds per superclass and class in investigated wastewater samples

Using WIE resins combined with a HLB sorbent as an extraction method that showed the best measured performance for the non-targeted analysis of wastewater effluents from our first objective, a non-targeted screening study of influents and effluents across six representative WWTPs in Canada was conducted in our second objective. A total of 811 compounds were tentatively identified across all samples at various levels of confidence as shown in Fig. 3. A total of 639 compounds were identified as probable structures (unambiguous high quality MS^2^ library match and/or additional context information) and 162 as tentative candidates (best match with at least one other potential candidate) based on the concept of levels of confidence of Schymanski et al. [25]. The complete list of identified compounds and their level of confidence are shown in the supplementary information (Annotation_table.csv). The compounds qualified as probable structures were mostly identified with high quality MS^2^ library matches with little to no ambiguity. Molecular networking and compound class scoring were also used to gather enough diagnostic evidence to qualify TPs and congeners of various families of compounds as probable structures. Relative standard deviations of spiked internal standards are shown in Table S9. Variations were monitored for influents and effluents as these two matrices can vary widely. Details about the normalization are given in the supplementary information.

#### Consumer product additives

The consumer product additives superclass contained 328 tentatively identified compounds as shown in Fig. 3. Surfactants and antiseptics accounted for more than half of these, with 171 contaminants, while 69 polymers and polymer additives, 46 personal care products and food additives, and 18 intermediates and reagents were also found. A total of 138 congeners of compounds containing repeating ethylene oxide units (EO) were tentatively identified. These contaminants, used as surfactants, have been previously observed in surface waters, urban runoffs, and WW effluents [3, 6, 38]. Linear alkyl sulfonic acids were also tentatively identified (in negative ionization). Contaminants originating from tire wear leachates such as diphenyl guanidine, ditolyl guanidine, dicyclohexylurea, benzotriazole, and benzothiazole were also tentatively identified in multiple samples. Contaminants typically originating from tire wear particle leachates have been a source of interest in multiple studies in northern countries [39–42]. However, neither the antiozonant 6PPD nor its transformation product 6PPD-quinone, which has been reported to induce acute mortality in coho salmon in river waters receiving stormwater urban runoff [43], was detected. The hindered amine light stabilizer 4-hydroxy-2,2,6,6-tetramethylpiperidine-1-ethanol, used in the fabrication of plastic and observed in a previous study [7], was detected in all samples along with its TPs 2,2,6,6-tetramethyl-1-piperidinol, resulting from the hydrolysis, and the carboxy acid TP hydroxy-2,2,6,6-tetramethylpiperidine-1-acetic acid [44]. Linear alkyl amines and amides were also detected in all samples. Alkylamidopropyl betaines, used in shampoo, were found in all samples as previously reported in surface waters [6, 7].

**Fig. 4:**
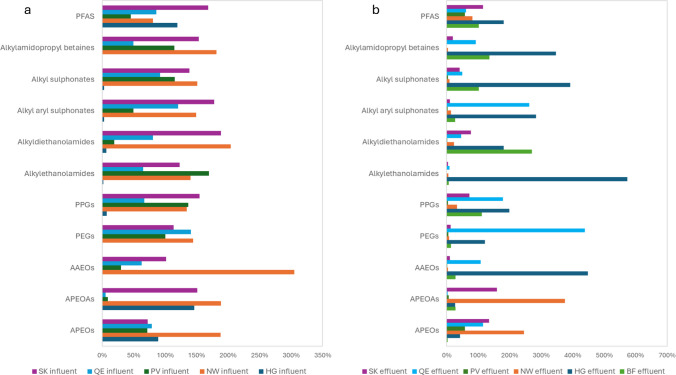
Relative average peak areas of compound families in WWTP influents (**a**) and effluents (**b**). PFAS, polyfluorinated alkyl substances; PPGs, polypropylene glycols; PEGs, polyethylene glycols; AAEOs, alkylalcohol ethoxylates; APEOAs, alkylphenol ethoxyacids; APEOs, alkylphenol ethoxylates

The relative peak areas of the main compound families are shown in Fig. 4. Despite compounds with higher peak not necessarily being present at higher concentrations, peak areas can be valuable for environmental risk assessors regarding prioritization of compounds. Alkylphenol ethoxylates were found in high relative intensity in NW influents and effluents, though the relative lower intensities of other families in the NW effluents could be explained by longer system hydraulic retention times (Table S1). The PV effluents exhibited vastly lower relative intensities as well, which can once again probably be explained by the longer system hydraulic retention times of the WWTP ranging from 3 to six months (Table S1). The HG effluents, however, had the highest relative intensities for 6 families of compounds, which could be explained by the contribution of the landfill leachate to the effluents.

#### Pharmaceuticals

We identified 149 pharmaceutical compounds across the six WWTPs influents and effluents (Fig. 3), which we have classified according to their Anatomical Therapeutic Chemical (ATC) code. The cardiovascular system pharmaceuticals were the most represented group with 54 compounds, 28 of which were transformation products (TPs) while the nervous system class was the second largest by substance number. Among systemic anti-infective pharmaceuticals, parent compounds and TPs from five families of antibiotics were tentatively identified: cephalosporins, fluoroquinolones, macrolides, lincomycins, and sulfonamides. This can be a cause of concern when considering bioresistance since a higher occurrence of antibiotics leads to a wider distribution of antibiotic resistance genes [45, 46]. The alimentary tract and metabolism class included the anti-diabetics gliclazide, sitagliptin, and metformin. 

#### Other compounds

The natural products and metabolites superclass, which was divided into lipids, amino acids and peptides, nucleobases and nucleotides, plant metabolites, hormones and “other” metabolites—a catch-all category for otherwise nonclassified compounds—was the second largest with 308 compounds (Fig. 3). Recreational drugs included legal and illicit drugs in Canada.

### Data mining tools comparison for the identification of transformation products and congeners

During the data treatment in the non-targeted study, the ability of two data mining tools to identify transformation products and congeners of chemical mixtures was assessed while the drawbacks were investigated as well.

#### Molecular networking

**Fig. 5:**
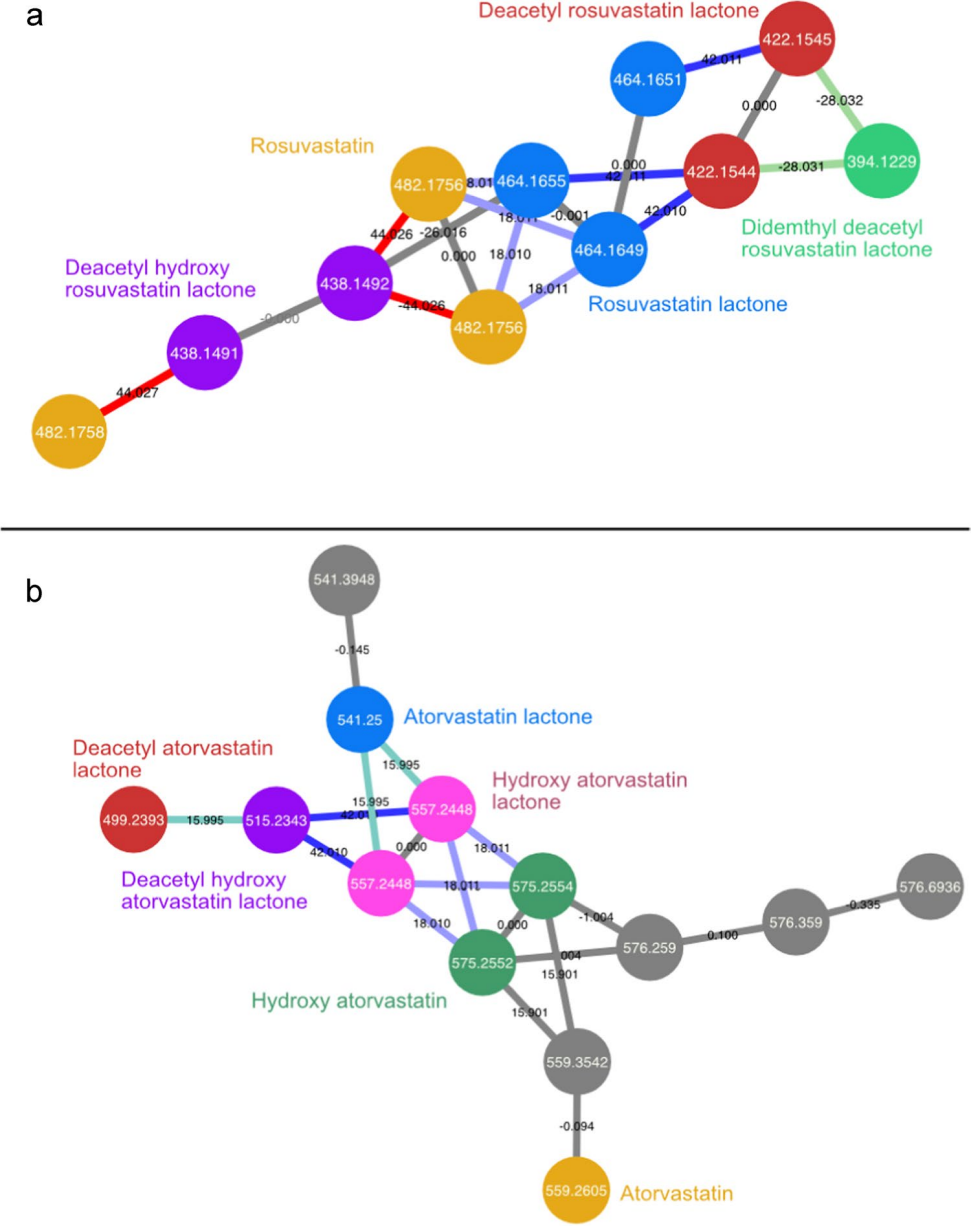
Molecular networks of the synthetic statins rosuvastatin (**a**) and atorvastatin (**b**). In gold are the untransformed parent compounds, in dark green the TPs following a hydroxylation, in blue is the lactone formation, in red the deacetylation and lactone formation, in purple the deacetylation, hydroxylation and lactone formation, in light green the didemethylation, deacetylation and lactone formation, and in pink the hydroxylation and lactone formation

For the identification of TPs and congeners in molecular networks, we first determined if the compounds identified with MS^2^ library matching with a high level of confidence were present in a network with multiple additional ions or were not linked with any ion. All the transformation products shown in Table 2 were found in this manner. For pharmaceuticals compounds, 37 transformation products were identified with molecular networking as clusters of beta-blockers, synthetic statins, calcium channel blockers, angiotensin II receptor antagonists, and their TPs were found (Table 2). Among synthetic statins, the rosuvastatin and atorvastatin networks, shown in Fig. 5, contained eleven identified TPs. Similar transformations, such as lactone formation and deacetylation, were observed on both rosuvastatin and atorvastatin. Only the untransformed compounds were identified with MS^2^ libraries while the TPs were all identified with molecular networks, showing how molecular networking can identify potential structural relationships that help overcome gaps in spectral database coverage. In the case of atorvastatin, all but one TP had higher apparent abundances than the parent compound. Several of the atorvastatin TPs have been identified in past studies, but some, such as deacetyl hydroxyatorvastatin lactone and deacetylatorvastatin lactone, have not been reported previously to our knowledge [38, 47]. This demonstrates the power of molecular networks as tools as they facilitate the direct identification of previously unreported TPs in complex samples. Furthermore, common metabolism transformations such as hydroxylation, dealkylation, deacetylation, and the loss of C_2_H_4_O moieties can readily be highlighted with edge annotation as shown in Fig. 5 where nodes are coloured in cyan, green, red, and navy blue, respectively. Detailed information on the compounds, transformation products, and congeners that were found in molecular networks referred to in Table 2 is given in Section 2.2 of the Supplementary Information.

**Table 2:** Transformation products and congeners classed by that were identified with molecular networking

Super class	Compounds	Number of transformation products and/or congeners identified	Figure
**Pharmaceuticals**	Diltiazem and TPs	5	Fig. S9
	Olmesartan and TPs	5	
	Irbesartan and TPs	5	Fig. S10
	Atorvastatin and TPs	4	Figure 5a
	Rosuvastatin and TPs	5	Figure 5b
	Multiple beta-blockers and TPs	6	Fig. S8
	Gliclazide and TPs	3	Fig. S13
	Clindamycin and TPs	4	Fig. S11
	**Total**	**37**	NA
**Recreational drugs**	Cocaine	1	Fig. S14
**Consumer product additives**	Tris(2-butoxyethyl)phosphate and TPs	6	Fig. S2
	Lauramidopropyl betaine and congeners	8	Fig. S5
	Lauryldiethanolamide and congeners	5	Fig. S6
	Laurylethanolamides and congeners	4	Fig. S7
	Nonylphenols ethoxylates congeners	10	Fig. S8
	Nonylphenols ethoxyacids congeners	6	Fig. S9
	Octylphenols ethoxyacids congeners	3	Fig. S9
	**Total**	**42**	NA

In general, a large majority of the TPs were identified through molecular networking. In these cases, no MS^2^ library matches were obtained. In several instances, for example with diltiazem, irbesartan, atorvastatin, the beta-blockers, gliclazide, and clindamycin, TPs of the pharmaceutical compounds were detected at similar or even higher intensity than their parent compounds. This can be of particular interest for risk assessment purposes as TPs can be still pharmacologically active and the concentration of the parent compound alone would not be enough information to predict its toxicity. Indeed, when assessing the removal of pharmaceutical compounds by wastewater treatments, the pharmacologically active TPs should be included in that evaluation.

A high number of consumer product additives were identified as congeners and homologues of large families of compounds. Molecular networking allowed ready identification of the alkylamidopropylbetaines, alkyldiethanolamides, and alkylethanolamides. In these cases, as with pharmaceuticals and their TPs, only one of the congeners was identified with a MS^2^ library match and all other ones were tentatively identified through molecular networking.

There were, however, shortcomings with molecular networking. Coeluting isobars in DDA mode within the isolation window (in this case 1 m/z) caused mixed MS^2^ spectra. This led to product ions being attributed to the wrong precursor ion mass which caused “imposter” nodes in molecular networks and complicated their uses as well as causing library matches with high mass error. This former phenomenon was particularly observable with gliclazide (Fig. S13) and cocaine (Fig. S14) networks and, to a lesser degree, with atorvastatin (Fig. 5b). Additionally, the collision energy needed to obtain adequate MS^2^ spectra ranged dramatically. In the MS^2^ spectra of linear alkyl sulfonic acids, the precursor ion remained by far the most intense ion, indicating that a higher collision energy would have been needed. Conversely, the vast majority of the compounds had high-quality MS^2^ spectra. Stepped collision energy, while intended to resolve this issue, has shown to lead to fewer library matches and less complete molecular networks [33]. Another major drawback of molecular networking lies in the reliance on a high number of common fragment ions—at least 3 or 4—that might not be present in the MS^2^ spectra of all compounds and their prospective TPs or congeners. These shared spectra being the key feature in creating molecular networks means smaller compounds or compounds with only one major fragment ion are not linked in molecular networks. This issue was also observed for 16 pharmaceuticals and their TPs. All these TPs were identified with MS^2^ library matches as none was found in a molecular network. In cases where they would not be registered in MS^2^ libraries, these would not have been tentatively identified. Furthermore, a higher signal intensity for both the parent compound and its TP did not mean that both compounds were linked together in a molecular network; despite acetaminophen being one of the compounds with the highest peak areas, it was not found in a network with its TP 4-acetamidophenol. Neither were venlafaxine and O-desmethylvenlafaxine, both very intense ions and commonly found in WW effluents and surface waters [3, 48–50].

#### Compound class scoring

The compound class scoring (CCS) tool in the Compound Discoverer software was used to screen expected families of contaminants based on previous studies of wastewater and surface water samples directly downstream of wastewater treatment plants [3, 6, 7]. Common fragment ions from linear alkylalcohol ethoxylates (AAEOs) and alkylphenol ethoxylates (APEOs) were manually entered for the corresponding respective compound classes that are shown in the Supplementary Information (Table S7 and Table S8). The AAEOs subclass is composed of families of congeners named according to the length of the linear alkyl chain of the congeners while the number of EO units varies in the same family; the C10E family, for example, has a linear decyl chain with varying numbers of EO units.

**Fig. 6:**
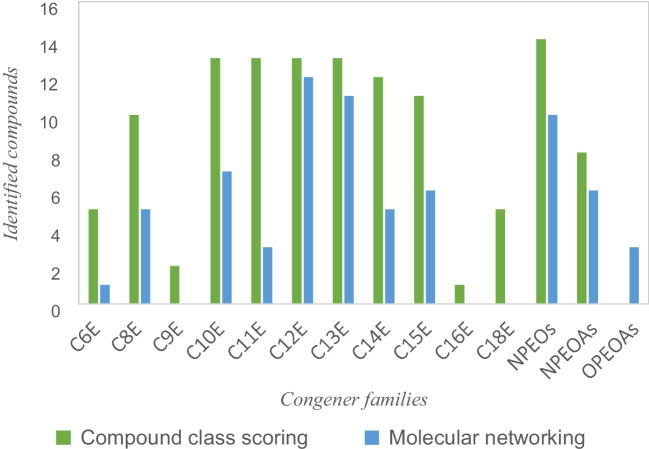
Number of ethylene oxide mixtures congeners identified by compound class scoring and molecular networking per family of congeners in investigated wastewater samples. The C6E nomenclature refers to an alkyl chain of 6

A total of 138 oligomers containing EO units were readily identified with Compound Class scoring. Of these EO oligomers, 100 were AAEOs, 15 were polyethylene glycols, 15 were nonylphenol ethoxylates, and 8 were their TPs nonylphenol ethoxylate acids. As shown in Fig. 6, twice as many AAEO congeners were tentatively identified with CCS than with molecular networking. Moreover, CCS is simpler to use by sorting features by spectra overlap, i.e., the features with the most fragment ions in common with the compound class, which allows ready annotation of AAEO families. The difference between CCS and molecular networking was less marked with APEO congeners and their transformation products, alkylphenol ethoxyacids. The use of DIA meant that MS^2^ spectra for less intense ions that might not have been selected for DDA MS^2^ acquisition were obtained, which highlights its ability to complement DDA.

**Fig. 7:**
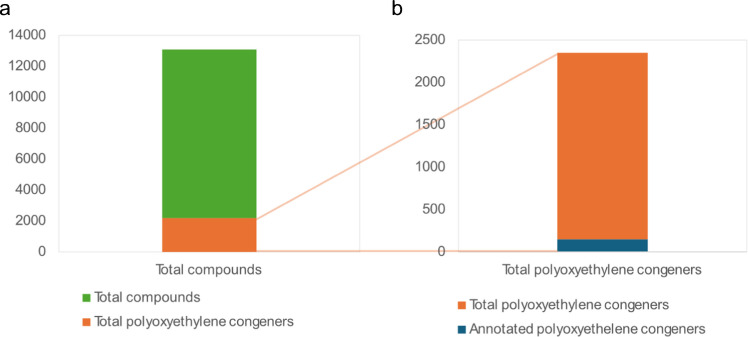
**a** Polyoxyethylene congeners detected vs total number of compounds detected in the positive electrospray mode. **b** Polyoxyethylene congeners tentatively identified vs polyoxyethylene congeners detected in investigated wastewater samples

Despite the usefulness of CCS and molecular networking, only a fraction of the total detected polyoxyethylene congeners were identified. In electrospray positive ionization mode, the ionization mode in which these contaminants are detected at the largest intensity, over 2200 unique compounds, ranging from 166 to 2447 Da, contained two or more fragment ions common to polyoxyethylene compounds in their MS^2^ spectra. Considering that 10,878 unique compounds were detected in the positive mode, this means that around 20% of all detected compounds were identified as ethylene oxide (EO) congeners, of which less than 7% were annotated as shown in Fig. 7. The structures for a significant number of these compounds are unknown as the structures of congeners from numerous technical mixtures are trade secrets [1]. Figure 7a shows a stark picture of the challenge when it comes to qualifying the polyoxyethylene contamination in wastewater samples and that they represent a significant part of the > 350,000 contaminants and mixtures on the market [1, 18].

### Wastewater treatment types and impact on contaminant profiles

The decrease in peak areas of the contaminants when comparing influents and effluents of a given WWTP was correlated with the system hydraulic retention time (Table S1). Such a decrease due to the treatments was, for example, not observed for QE WWTP, which had the lowest retention time. Influents from WWTP QE had low relative intensities compared to the other influents while the QE effluents showed high relative intensities compared to the other effluents. The use of secondary biofilters instead of secondary activated sludge, as was the case in the similarly sized SK, could also be a factor. In the NW facultative lagoon, polyoxyethylene compounds were also detected with by far the highest intensity in this WWTP as seen in Fig. 4. The shorter chain nonylphenol ethoxylates and the nonylphenol ethoxyacids, all TPs of longer chain nonylphenol ethoxylates, were notably detected at high intensities in the NW effluents, suggesting that transformation occurred in the lagoon. This can highlight additional environmental concerns because of the estrogenic activity of these contaminants in transformation [51].

## Conclusion

The comparative assessment of mixed mode weak and strong ion exchange cartridges with hydrophilic-lipophilic balance resins filled the information gap on the best practices for SPE extraction of WW effluents for non-targeted analyses. Then, as state-of-the-art data mining tools for non-targeted and suspect screening were evaluated, molecular networking was effective for the identification of transformation products as well as congeners from multiple smaller families while CCS particularly excelled at detecting large families like ethylene oxide (EO) congeners.

This study highlights the ability to characterize organic contaminants in WWTP effluents and influents, especially TPs and congeners, while also highlighting the number of unknown congeners present. The approaches evaluated in this study allowed progress from unknown unknowns to known unknowns for numerous congeners such as EO congeners. This represents a path toward characterizing the sheer mass of organic loadings released in the aquatic environment, and indicating which contaminants require more extensive assessment.

## Supplementary Information

Below is the link to the electronic supplementary material.

Supplementary file1 (DOCX 5.93 MB)

Supplementary file2 (CSV 80.4 KB)

## Data Availability

Data published in the manuscript and additional data can be requested from the corresponding author.
