# Supplementary material for: Assessing the performance of weak and strong ion exchange solid-phase extraction and data mining tools to identify congeners and transformation products in municipal wastewaters by non-targeted analysis
Source: Anal Bioanal Chem. 2026 Mar 25;418(11):3195–209. doi: 10.1007/s00216-026-06423-3 (PMC13197246; doi:10.1007/s00216-026-06423-3)
Supplement: Supplementary file 1 — Supplementary file1 (DOCX 5.93 MB) [file 216_2026_6423_MOESM1_ESM.docx]

Assessing the performance of weak and strong ion exchange solid phase extractions and data mining tools to identify congeners and transformation products in municipal wastewaters by non-targeted analysis – Supplementary Information

Emmanuel Eysseric^1^, Christian Gagnon^1^, L. Mark Hewitt^2^, Shirley Anne Smyth^3^.

^1^: Aquatic Contaminants Research Division, Environment and Climate Change Canada, 105 McGill St., Montreal, QC Canada

2: Aquatic Contaminants Research Division, Environment and Climate Change Canada, 867 Lakeshore Rd., Burlington, ON Canada

3: Regulatory Operations, Policy, and Emerging Sciences Division, Environment and Climate Change Canada, 867 Lakeshore Rd., Burlington ON Canada

# Materials and methods

## Wastewater treatment plants

**Table S1.** WWTP characteristics and sampling dates

| **WWTP type** | **Aerated**  **Lagoon**  **PV** | **Facultative Lagoon with Filters**  **NW** | **Secondary Biofilter**  **QE** | **Secondary Extended Aeration**  **HG** | **Secondary Activated Sludge SK** | **Secondary Activated Sludge**  **BF** |
| --- | --- | --- | --- | --- | --- | --- |
| **Samples** | Influents and effluents | Influents and effluents | Influents and effluents | Influents and effluents | Influents and effluents | Effluents only |
| **Sampling method** | Grab | Grab | Composite | Composite | Composite | Composite |
| **Average flow (m^3^/day)** | 400 | 800 | 161,460 | 2,050 | 139,740 | 43,540 |
| **% Residential inputs** | 90 | 100 | 90 | 90 | 50 | 45 |
| **% Industrial-Commercial-Institutional inputs** | 10 | 0 | 10 | 10 | 50 | 55 |
| **Significant industries** | Sandpaper company | Beef processing | Various | Dairy Company, landfill leachate | Various | Chemical production, landfill leachate |
| **System hydraulic retention time** | 3 to 6 months | 3 months | 45 mins | 27 hrs | 12 hrs | 30 hours |
| **Operated to nitrify?** | Yes | Yes | No | Yes | Yes | Yes |
| **Phosphorus removal** | Alum | Alum | No | Alum | Ferric Chloride | Ferric Chloride |
| **Tertiary filtration** | Sand filters | Sand filters | No | Sand filters | Sand filters | No |
| **Disinfection** | No | No | Ultraviolet May to Oct | Ultraviolet | Ultraviolet Apr to Oct | Hypochlorite with de-chlorination (year round) |
| **Sampling dates** | 24-26 Oct 2023 | 11-13 Apr 2023 | 20-22 Jun, 2023 | 9-11 May 2023 | 15-17 Aug 2023 | 4-6 Jun, 2024 |
| **Influent temperature range (°C)** | 16 to 17 | 10 to 12 | 14 to 15 | 12 to 15 | 20 to 22 | 17 |
| **Effluent**  **temperature range (°C)** | 11 to 13 | 12 to 14 | 16 to 17 | 12 to 13 | 19 to 21 | 23 to 26 |
| **Solids retention time (d)** | N/A | N/A | N/A | 20 – 24 | 11.5 | 8 |

## Extraction

**Table S2.** Sampling, purification and extraction methods and techniques in investigated non-targeted studies

| **Matrix** | **Sampling, purification and extraction** | **Reference** |
| --- | --- | --- |
| Marine Water (urban runoff, agricultural discharge, WW effluent) | POCIS (HLB) for 3 weeks, 10 MTBE in MeOH for elution | [1] |
|  | Grab samples, 0.45 um GF/F, HLB 500 mg, spiked with internal standards prior to extraction, 10% MTBE in MeOH for elution |  |
| WW | Flow-weighted 24 hours composites, 0.45 um GF/F, HLB 500 mg, spiked with internal standards prior to extraction, 10% MTBE in MeOH for elution |  |
| WW effluent | Grab samples, WAX (150 mg), HLB (500 mg), WCX (150 mg) | [2] |
| Industrial WW and | Grab samples, 0.45 um glass microfiber, pH adjusted to 8 with NH_4_OH, HLB 200 mg | [3] |
| Urban WW |  |  |
| Surface water, marine water and WW | Grab samples and 24 h composites, 0.45 um GFF and PVDF filtration, pH 6.5, Strata X, X-AW, X-CW, Isolute ENV+ | [4] |
| Sewage and WW treatment plants effluents (urban and industrial) | HLB (200 mg), Strata-XAW (100 mg), Strata-XCW (100 mg), Isolute ENV+ (150 mg) | [5] |
| WW | Grab samples, modified QuEChERS method, | [6] |
| WW | organic-diffusive gradients in thin-films (o-DGT) and POCIS, 24 h composites, HLB | [7] |
| Hospital WW | 0.45 um cellulose, HLB (60 mg) | [8] |
| Surface water, sediments and WW | HLB (200 mg), MeOH-CH_2_CL_2_ for elution | [9] |
| WW | Waters BEH cartridges (500 mg), | [10] |
| WW | 24 h composite, 6.5 pH adjusted, HLB (200 mg), Isolute ENV+ (150 mg), Strata-XAW (100 mg), Strata, X-CW (100 mg) in one cartridge | [11] |
| WW (chemical and urban) | Grab samples and 4 h composites, HLB (500 mg), | [12] |


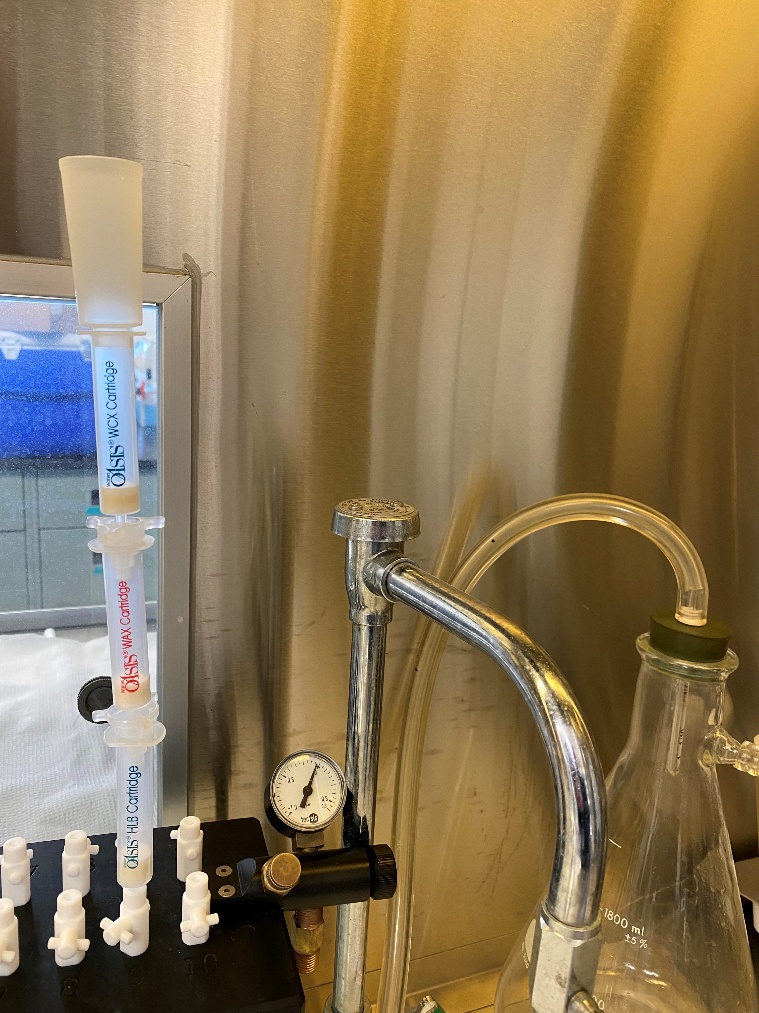


**Figure S1.** Set up in series of solid phase extraction apparatus.

## Liquid chromatography

**Table S3.** Liquid chromatography gradient for the positive and negative electrospray ionization

| **Time (min)** | **%B** | **Flow rate (mL min^-1^)** |
| --- | --- | --- |
| 0 | 0 | 250 |
| 3 | 0 | 250 |
| 25 | 100 | 250 |
| 30 | 100 | 250 |
| 30.01 | 0 | 400 |
| 36 | 0 | 400 |

## Mass spectrometry

### Source parameters

For positive ion mode, the instrument was operated with a sheath gas flow rate of 50, auxiliary gas flow of 20, and a sweep gas flow rate of 1 mL min^-1^. The auxiliary gas temperature was maintained at 400°C, while the capillary temperature was set at 300°C. The spray voltage was adjusted to 3.5 kV, and the S-lens RF level was set to 50 V. For negative ion mode, the sheath gas flow rate remained at 50, with the auxiliary gas flow rate of 20, and the sweep gas flow rate at 1 m min^-1^. The auxiliary gas temperature was kept at 400°C, and the capillary temperature was 300°C. The spray voltage was set to 3.4 kV, with the S-lens RF level maintained at 50 V.

### Mass spectrometry acquisition modes

#### Full scan

Scan range was 100 to 1200 m/z, AGC Target was 1e6, miscroscans were set to 1 and Ion Injection Time was set to 100 ms.

#### Data dependent acquisition

The Data Dependent Acquisition (DDA) settings were selected based on the study of Q-Exactive parameters for optimal library identification and molecular networking formation by Stincone et al.[13] The DDA files were used for identification purposes with HRMS^2^ spectra database as well as for molecular networks. A top K of 3 was used with apex trigger and dynamic exclusion in order to get a good compromise between a fast enough frequency of acquisition and enough MS^2^ spectra. The scan range was set from 100 to 1200 m/z. The automatic gain control (AGC) target was adjusted to 1e5, with the apex trigger enabled. Dynamic exclusion was activated to prevent repeated analysis of the same ions. An isolation width of 1 m/z was used, and isotope exclusion was turned on to exclude isotopic peaks. A single microscan was performed per scan, and monoisotopic selection was enabled to prioritize monoisotopic peaks. The ion injection time was set to 55 ms, and the normalized collision energy (NCE) was fixed at 30. The top 3 most intense ions were selected for fragmentation in each scan cycle. The resolution of the mass spectrometer was set to 17,500.

#### Data independent analysis

Data-independent acquisition (DIA) is a type of tandem mass spectrometry experiment used to analyze complex mixtures of molecules. Unlike traditional methods that select specific ions for fragmentation, DIA simultaneously fragments all ions within a specified mass range. This approach allows for comprehensive and unbiased data collection, capturing a wide array of molecular information in a single run. The resulting data can then be analyzed to identify and quantify numerous compounds, providing a detailed overview of the sample’s composition. DIA generates more feature rich MS^2^ spectra. The scan range was set from 100 to 1200 m/z. The automatic gain control (AGC) target was adjusted to 5e5 to ensure sufficient ion accumulation. The ion injection time was set to 55 ms, and the normalized collision energy (NCE) was fixed at 30. The resolution of the mass spectrometer was maintained at 17,500 to achieve high mass accuracy. The acquisition windows were set to 50 Da.

### Internal standards

Internal standards were used to monitor the extraction and instrument performance as well as for data normalization and potential quantification or semi-quantification. Stable isotope standards already available in the laboratory inventory were used. The values in Log K_ow_, or logP, range from 0.6 to 2.9 or around four orders of magnitude in polarity proxy values as can be seen in Table S3.

**Table S4.** Spiked stable isotopes standards spiked prior to the extraction for the evaluation of the extraction methods

| **Compound** | **LogP** | **pKa** |
| --- | --- | --- |
| atrazine-d5 | 2.6 | 1.6 |
| Amitryptiline-d3 | 5 | 9.4 |
| Bupropion-d9 | 3.2 | 8.35 |
| capecitabine-d11 | 0.6 | 1.9 |
| cis-tramadol-13C-D3 | 2.6 | 9.41 |
| citalopram-d6 | 3.2 | 9.38 |
| cyclophosphamide-d4 | 0.6 | 8.22 |
| diazepam-d5 | 3 | 3.4 |
| Sertraline-d3 | 4.8 | 8.5 |
| venlafaxine-d6 | 2.9 | 10.09 |

**Table S5.** List of internal standards spiked prior to extraction for the non-targeted screening of the 6 WWTPs

| **Compound** | **Monoisotopic mass** | **LogP** | **pKa** |
| --- | --- | --- | --- |
| atrazine-D_5_ | 220.12515 | 2.6 | 1.6 |
| capecitabine-D_11_ | 370.21831 | 0.6 | 1.9 |
| cyclophosphamide-D_4_ | 264.04993 | 0.6 | 8.22 |
| Imatinib-D_3_ | 496.2778389 | 3.5 | 7.84 |
| Sulfamethoxazole-^13^C_6_ | 259.07224 | 0.9 | 1.6 |

**Table S6.** Nontargeted Analysis Study Reporting Tool for the current study

| **Section** | **Category** | **Sub-Category** | **Example Information to Report** | **Score (0-3)** |
| --- | --- | --- | --- | --- |
|  |  |  |  |  |
|  |  |  |  |  |
| [Methods](https://nontargetedanalysis.org/reference-content/methods/) | [Study Design](https://nontargetedanalysis.org/reference-content/methods/study-design/) | [Objectives & Scope](https://nontargetedanalysis.org/reference-content/methods/study-design/#objectives-and-scope) | • Study goals and hypotheses • Scope of the study with respect to use of NTA / suspect screening • Expected chemical coverage of approach and potential limitations | **3** |
|  |  | [Sample Information & Preparation](https://nontargetedanalysis.org/reference-content/methods/study-design/#sample-info-and-prep) | • Sample collection/replication, handling/storage, preparation, extraction, & clean-up methods (and related QA practices) • Intended use of samples (e.g., method development, compound identification, etc.) • Development and intended use of blanks | **3** |
|  |  | [QC Spikes & Samples](https://nontargetedanalysis.org/reference-content/methods/study-design/#qc-spikes-and-samples) | • Development of QC spikes/samples (e.g., isotopically labeled standards/spikes, native standard spikes, matrix pools) • Intended use of QC spikes/samples (e.g., to monitor instrument performance, data normalization, etc.) | **2** |
|  | [Data Acquisition](https://nontargetedanalysis.org/reference-content/methods/data-acquisition/) | [Analytical Sequence](https://nontargetedanalysis.org/reference-content/methods/data-acquisition/#analytical-sequence) | • Sample randomization and use of replicate injections • Inclusion of blanks and QC samples in the acquisition sequence • Information about single vs. multiple analytical batches | **2** |
|  |  | [Chromatography](https://nontargetedanalysis.org/reference-content/methods/data-acquisition/#chromatography) | • Instrument specifications • Method settings (e.g., column/guard, mobile phases, gradient, injection techniques) | **3** |
|  |  | [Mass Spectrometry](https://nontargetedanalysis.org/reference-content/methods/data-acquisition/#mass-spec) | • Instrument specifications • Instrument calibration and/or tuning procedures • Method settings (e.g., acquisition parameters, such as polarity, resolution, data-dependent vs. data-independent) | **3** |
|  | [Data Processing & Analysis](https://nontargetedanalysis.org/reference-content/methods/data-processing-and-analysis/) | [Data Processing](https://nontargetedanalysis.org/reference-content/methods/data-processing-and-analysis/#data-processing) | • File conversion information (e.g., to open-source format, centroiding) • Software program(s) used • Workflow steps (e.g., peak picking, RT calibration, alignment, gap filling) and settings • Feature detection thresholds (e.g., replicate detection criteria; min height, area, or S/N levels; comparison to occurrence/abundance in blanks) • Data correction or normalization methods (e.g., peak area/height normalization or scaling, blank subtraction) | **3** |
|  |  | [Statistical & Chemometric Analysis](https://nontargetedanalysis.org/reference-content/methods/data-processing-and-analysis/#statistical-analysis) | • Software programs(s)/package(s) used & samples/sample groups to which analyses were applied • Basic statistical analysis method goals (e.g., summarize data, evaluate variability, hypothesis testing), type (e.g., Wilcoxon rank sum test, Chi-square test), assumptions, and settings/thresholds • Chemometric analysis method goals (e.g., prioritize features, compare/classify samples, evaluate relationships between features), type (e.g., differential analysis, hierarchical clustering, dimensionality reduction), assumptions, and settings/thresholds | **3** |
|  |  | [Annotation & Identification](https://nontargetedanalysis.org/reference-content/methods/data-processing-and-analysis/#annotation-and-id) | • Software program(s) used (or description of manual annotation/identification efforts) • Libraries and databases used (including details such as chemical coverage, resolution, metadata inclusion; information about in-house databases) • Workflow steps (e.g., formula assignment, suspect screening, MS/MS spectral interpretation or library matching) • Workflow methods & settings (e.g., formula prediction method, scoring algorithms; mass error/RT tolerances, accepted match scores) | **3** |
| [Results](https://nontargetedanalysis.org/reference-content/results/) | [Data Outputs](https://nontargetedanalysis.org/reference-content/results/data-outputs/) | [Statistical & Chemometric Outputs](https://nontargetedanalysis.org/reference-content/results/data-outputs/#stats-out) | • Basic statistical outputs (e.g., adj. p-values, standard deviations, test statistics) • Results of chemometric analyses (e.g., reported classifications/groupings of features or samples, observed trends in the data) • Visuals/plots (e.g., Venn diagrams, heatmaps, clustering dendrograms, volcano plots, network diagrams, PCA and loading plots) • New statistical metrics, algorithms, packages, and/or scripts | **2** |
|  |  | [Identification & Confidence Levels](https://nontargetedanalysis.org/reference-content/results/data-outputs/#id-and-conf) | • Reported identifications and associated confidence levels (e.g., levels described by Schymanski et al., *ES&T*, 2014) • Supporting data for annotation/identification (e.g., formula match scores, fine isotope pattern, retention time match, MS/MS match scores, source of MS/MS spectra) • For features with lower confidence IDs, (i.e., not standard-confirmed), proposed tentative structures and other annotated data • Semi-quantification or quantification data • Exported MS/MS spectra (e.g., as a library, database, or deposition into online repository) | **2** |
|  | [QA/QC Metrics](https://nontargetedanalysis.org/reference-content/results/qa-qc-metrics/) | [Data Acquisition QA/QC](https://nontargetedanalysis.org/reference-content/results/qa-qc-metrics/#data-acq-qaqc) | • Quality: Adherence to QA/QC protocols for sample preparation and data acquisition • Boundary: Description of the potential impacts of methods (sample prep, chromatographic, MS) on observable chemical space • Accuracy: Reported chromatographic and mass accuracy • Precision: Variability of observed retention time, precursor mass error, and abundance | **2** |
|  |  | [Data Processing & Analysis QA/QC](https://nontargetedanalysis.org/reference-content/results/qa-qc-metrics/#data-proc-qaqc) | • Quality: Outcomes of QC checks along the data processing & analysis workflow • Boundary: Impact of data processing & analysis method(s) on observed chemical space, observed limits of detection/ID • Accuracy: Performance measures (True Positive Rate, False Positive Rate, etc.) for known compounds or samples with known classification • Precision: Reproducibility/repeatability of performance measures for known compounds or samples with known classification; Calculations such as False Discovery Rate, F1 score, etc. | **2** |

## Data analysis, treatment and visualization

Multiple workflows were used for data analysis and treatment. The Compound Discoverer software was used in all analysis types (full scan, DDA, DIA) for feature detection, identification and statistical analysis. MzMine was used with the DDA data for feature selection before using the Feature Based Molecular Networking on the Global Natural Product Social Molecular Networking website ([www.gnps.ucsd.edu](http://www.gnps.ucsd.edu) ) to generate molecular networks. Cytoscape was used for networks visualization. The graphs were created with Origin 2022 and Microsoft Excel. Affinity Designer 2 was used for further graphical modifications for figure design.


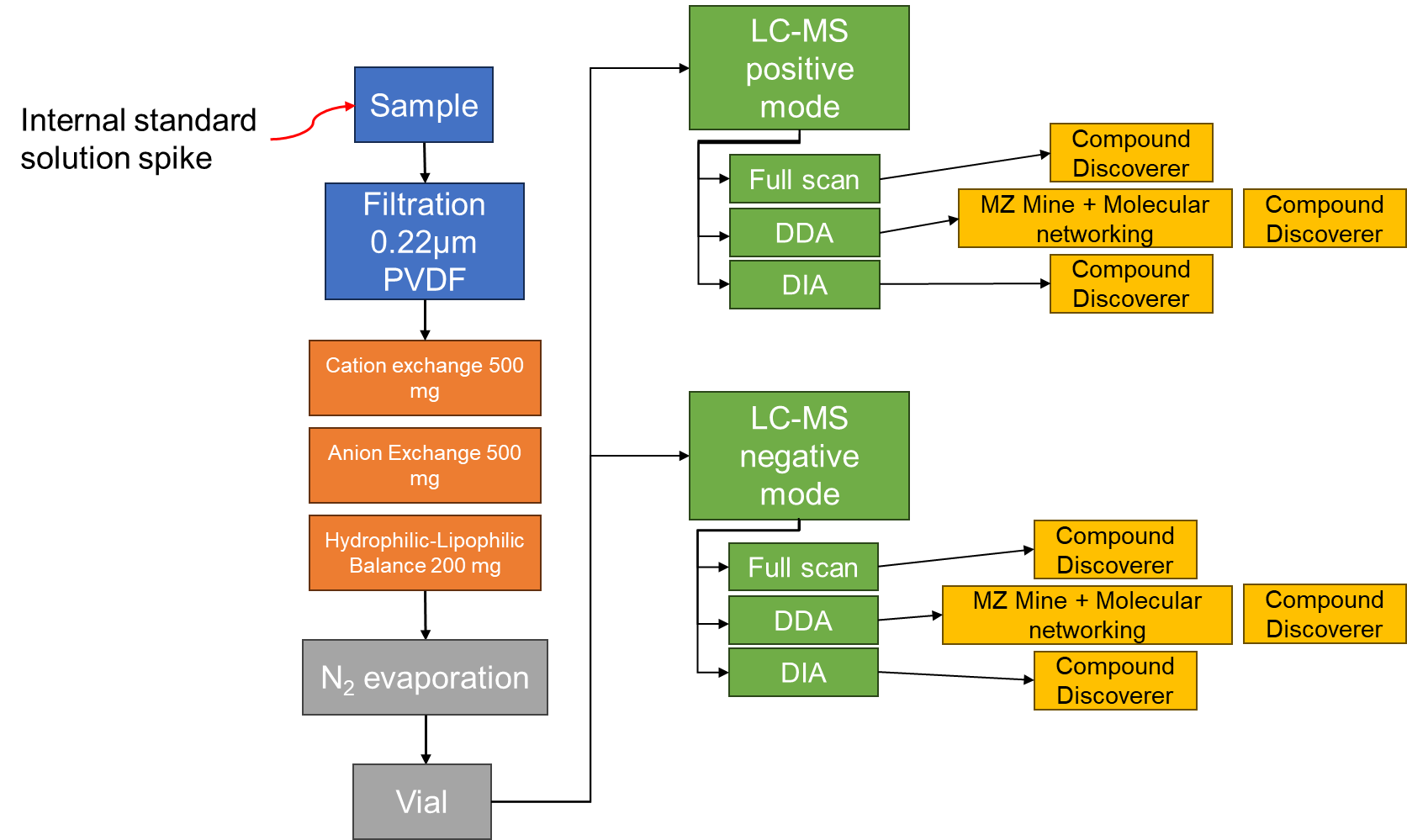


**Figure S2.** Schematic workflow of the study

**Table S7.** List of fragment ions for the compound classes used for the nonylphenol ethoxylates compound class scoring tool. *Print capture from Compound Discoverer.*


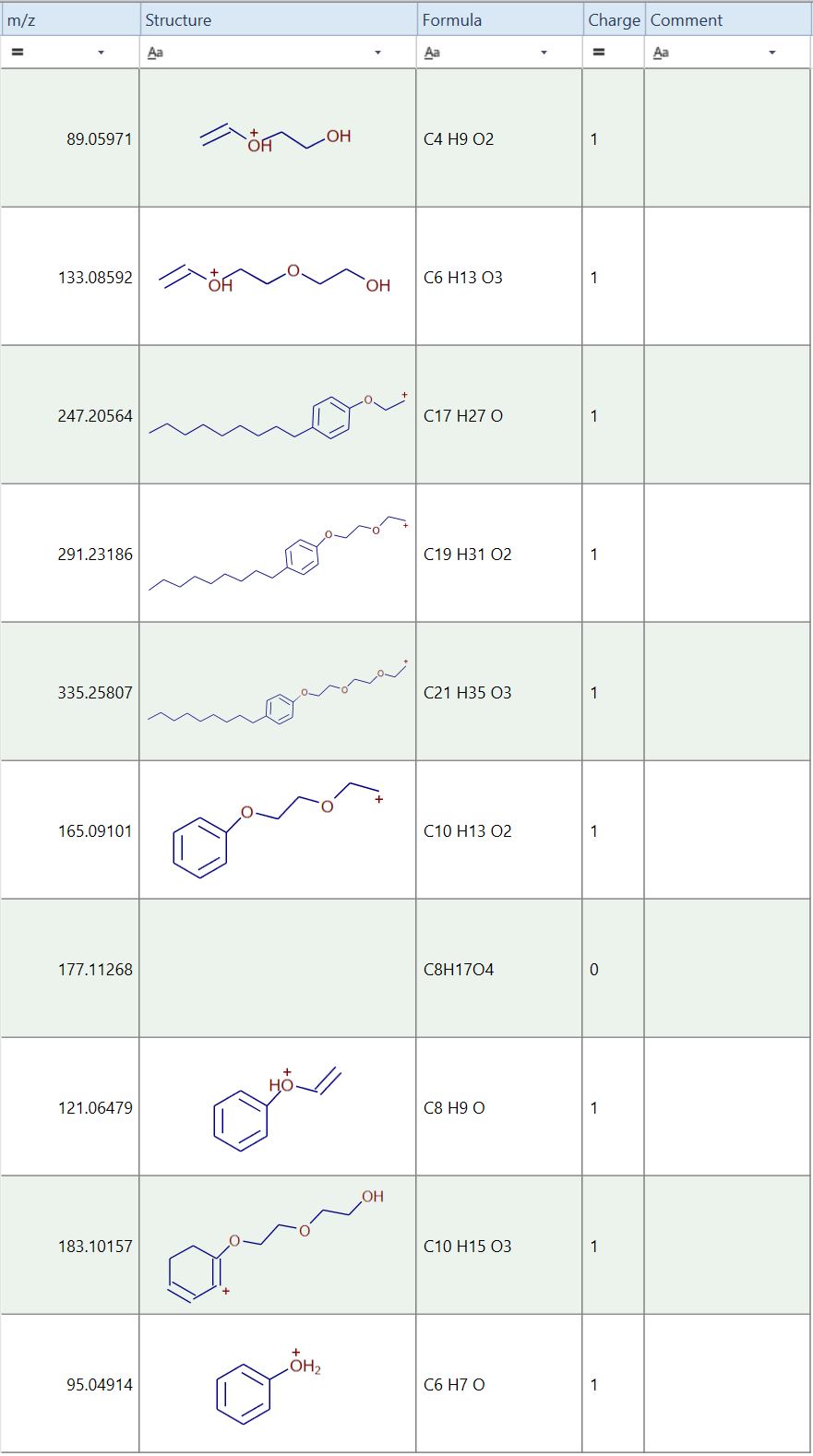


**Table S8.** List of fragment ions for the compound classes used for the polyethylene glycol compound class scoring tool. *Print capture from Compound Discoverer.*


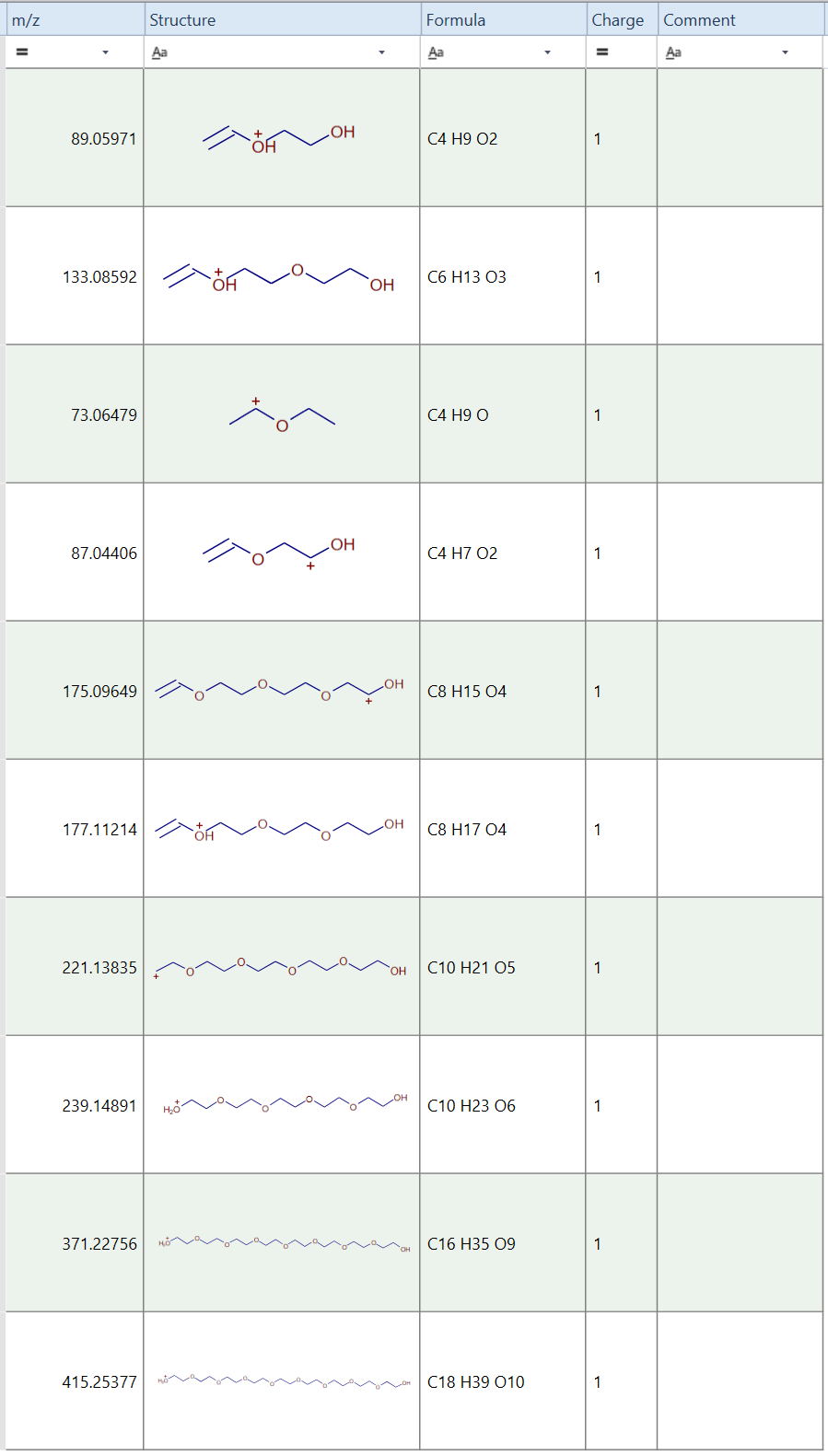


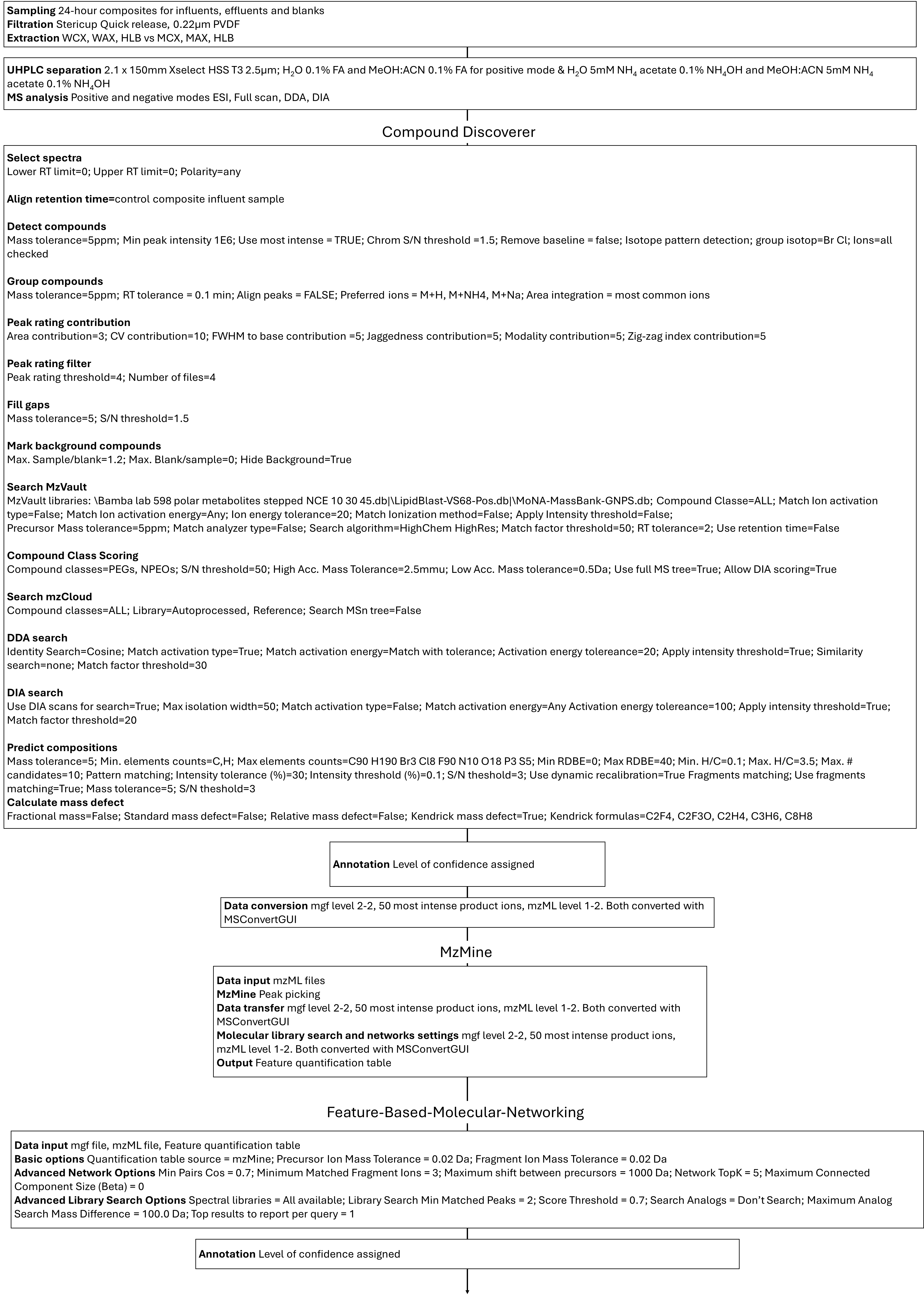


**Figure S3.** Workflow of the study and data treatment and processing settings

# Results

## Quality control

**Table S9.** Relative standard deviations for influents and effluents per internal standard

| Compound | Influents RSD | Effluents RSD |
| --- | --- | --- |
| Atrazine-D_5_ | 78% | 195% |
| Capecitabine-D_11_ | 161% | 167% |
| Cyclophosphamide-D_4_ | 276% | 214% |
| Imatinib-D_3_ | 135% | 108% |
| Sulfamethoxazole-^13^C_6_ | 322% | 277% |

Normalization for the families of compounds shown in Figure 4 was performed for influents and effluents separately.[14]

## Solid phase extraction optimization

**Table S10.** Fold change of individual compounds detected in the WIE over SIE extracts

| Superclass | Fold change WIE/SIE under 0.66 | Fold change WIE/SIE between 0.66 and 1 | Fold change WIE/SIE between 1 and 1.5 | Fold change WIE/SIE over 1.5 |
| --- | --- | --- | --- | --- |
| Consumer product additives | 19.34% | 14.36% | 41.44% | 22.65% |
| Natural products and metabolites | 34.65% | 20.08% | 32.28% | 12.99% |
| Pharmaceuticals | 21.93% | 12.28% | 54.39% | 11.40% |
| Recreative drugs | 5.88% | 5.88% | 52.94% | 35.29% |

## Data mining tools for the identification of transformation products and congeners

Multiple networks of pharmaceutical compounds and their transformation products were tentatively identified; 6 beta-blockers and 6 of their TPs were annotated in a network (**Figure S8**). Two other families of cardiovascular drugs were found in molecular networks with multiple transformations: the calcium channel blocker diltiazem and the angiotensin II receptor antagonist irbesartan. In the former’s case, it was found along with 7 TPs, 5 of which were tentatively identified (**Figure S9**). These compounds were the result of demethylation and deacetylation, which have been documented as the metabolism pathway of the parent compound; of note is that the TPs remain pharmacologically active.[15–17] These compounds have also been detected in surface waters in previous studies.[18, 19]

Only diltiazem and deacetyldiltiazem were identified with MS^2^ library matches; the other three aforementioned compounds were tentatively identified with molecular networking. We were not able to identify with confidence the other two TPs of diltiazem. A molecular rearrangement would happen in these compounds as the loss of the sulfur atom is observed considering the mass defect of the compounds. The relationship between the two unknown TPs would be then a demethylation. The second network featuring irbesartan and five of its TPs is shown in **Figure S10**. Four of the TPs were tentatively identified with molecular networking. Hydroxylation was the most commonly observed transformation with hydroxyirbesartan being by far the transformation product with the highest peak area while dihydroxyirbesartan also had a significant peak area The other TPs were irbesartan aldehyde, which was the result of the oxidation of hydroxyirbesartan and despropylirbesartan with the loss of propyl group. Finally, irbesartan_TP473 remained unannotated. These TPs, with the exception of irbesartan_TP473 which had not been reported, have been found previously in aqueous matrices. [18–21]

The larger macrolides antibiotics azithromycin and clarithromycin were found in a molecular network shown in **Figure S11** along with two other related compounds that remained unannotated. Furthermore, the lincomycin clindamycin was also detected in a molecular network with four TPs as shown in **Figure S12**. Three of the TPs, clindamycin sulfoxide, N-demethylclindamycin sulfoxide, and N-demethylclindamycin, were tentatively identified with MS^2^ library matches while the other, clindamycin_TP439, could be the result of the hydroxylation of clindamycin followed by an oxidation of the alcohol into an aldehyde or a ketone. Gliclazide and 3 transformation products were part of a molecular network shown in **Figure S13**. Hydroxy gliclazide and carboxy gliclazide were detected at much higher peak areas than gliclazide itself while the peak area of gliclazide aldehyde was lower. The recreative drug cocaine and its metabolites benzoylecgonine, that were identified with MS^2^ library matching tools, were found in a molecular network alongside the metabolite norbenzoylecgonine (**Figure S14)**. For consumer products additives, 6 transformation products of Tris(2-butoxyethyl)phosphate were identified following hydrolysis, hydroxylation and oxidation reactions (Figure S-2). Some of the TPs had been previously detected in past studies generated through laboratory controlled degradation or in the environment, but two novel TPs were discovered.[18, 22, 23] Furthermore, 17 congeners of personal care products alkylamidopropyl betaine, lauryldiethanolamide and laurylethanolamide, used in shampoos, were identified and are shown in Figures S-5, S-6 and S-7 respectively. Multiple polyfluorinated compounds were found in a very large molecular network (**Figure S3)**. There are multiple instances of duplicates among the nodes in this network and fewer individual compounds than what is shown. Still, the absence of a library match for any compound rendered their identification with a good level of confidence challenging. **Figure S4** shows a much smaller molecular network featuring two polyfluorinated acids, 7-tridecafluoroenanthic acid and 8-pentadecafluorooctanoic acid.

## Captures of the molecular networks


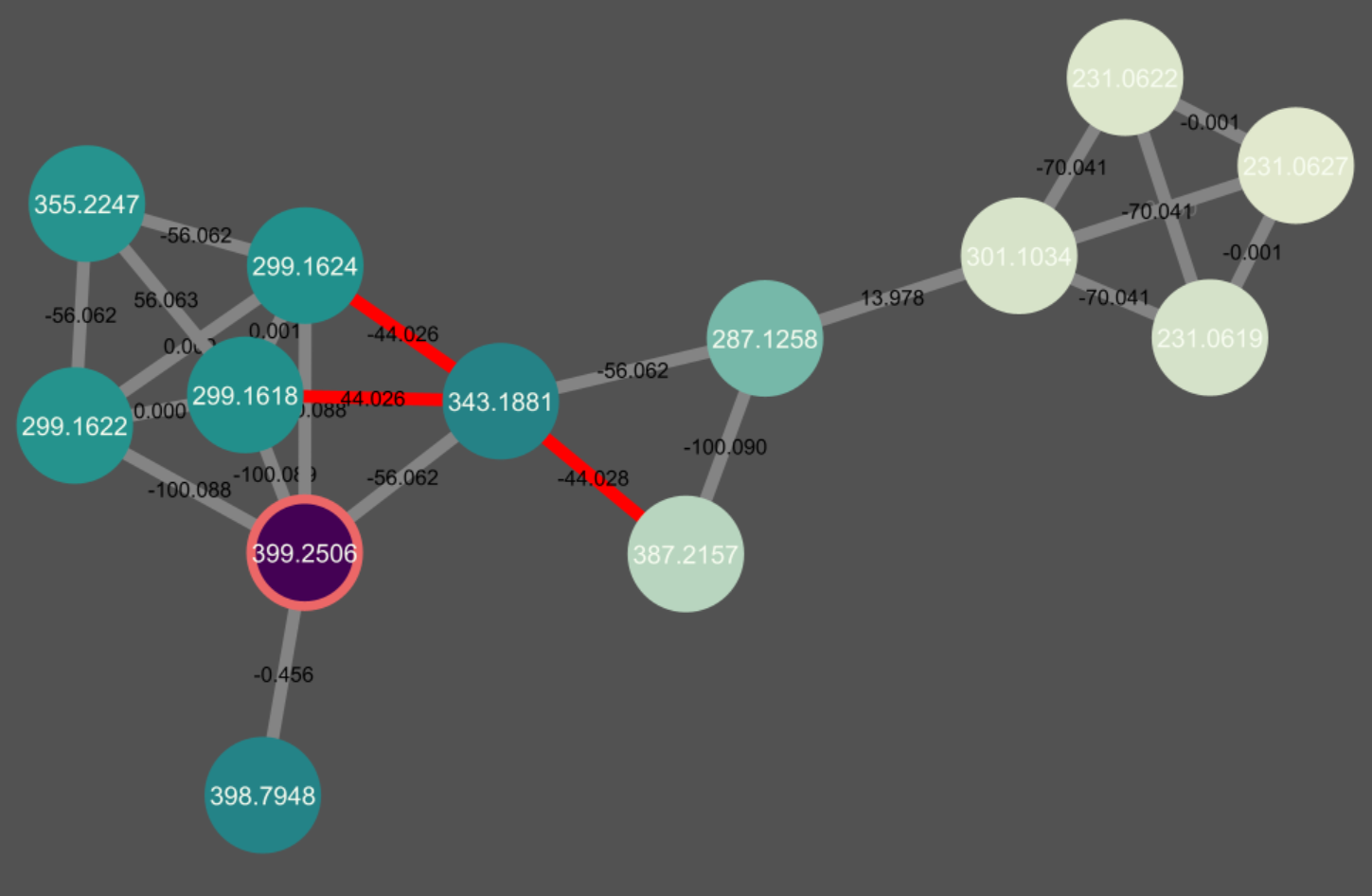


**Figure S5.** Molecular network of Tris(2-butoxyethyl) phosphate and its TPs. 287.1258 is TBEP_TP287; 299.1618, 299.1622, and 299.1624 are all Bis(2-butoxyethyl) phosphate; 301.1034 is TBEP_TP301; 343.1881 is TBEP_TP343; 355.2247 is TBEP_TP355; 387.2158 is TBEP_TP387; and 399.2506 is Tris(2-butoxyethyl) phosphate. A structure for 231.0622 could not be determined while 398.7948 is a coeluting isobar of Tris(2-butoxyethyl) phosphate.


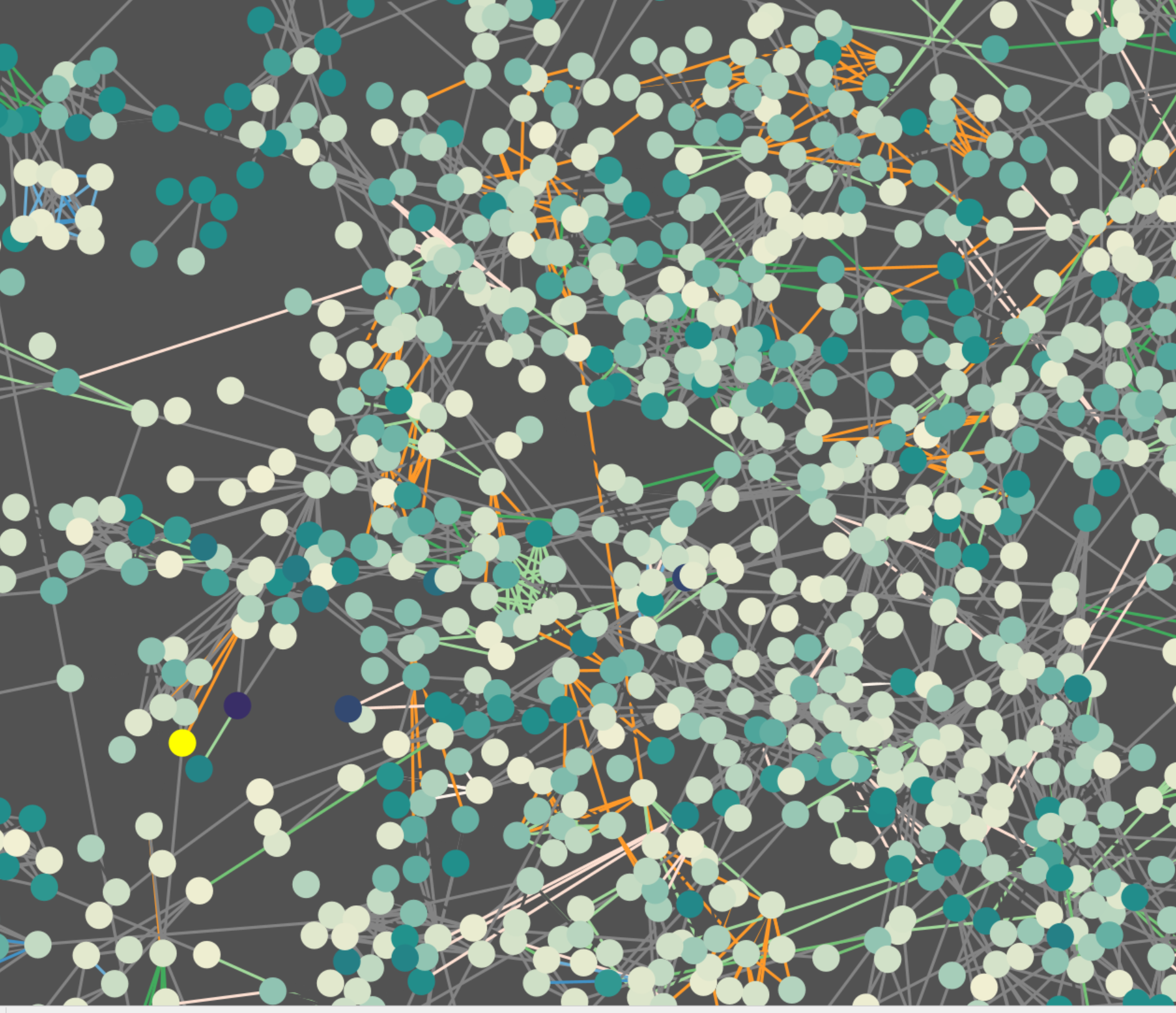


**Figure S6.** Close up on a large molecular network containing multiple polyfluorinated unidentified compounds. Nodes linked with an orange line denote a m/z difference corresponding to the loss or gain of a CF_2_ unit. As such, compounds linked with an orange line would be polyfluorinated compounds.


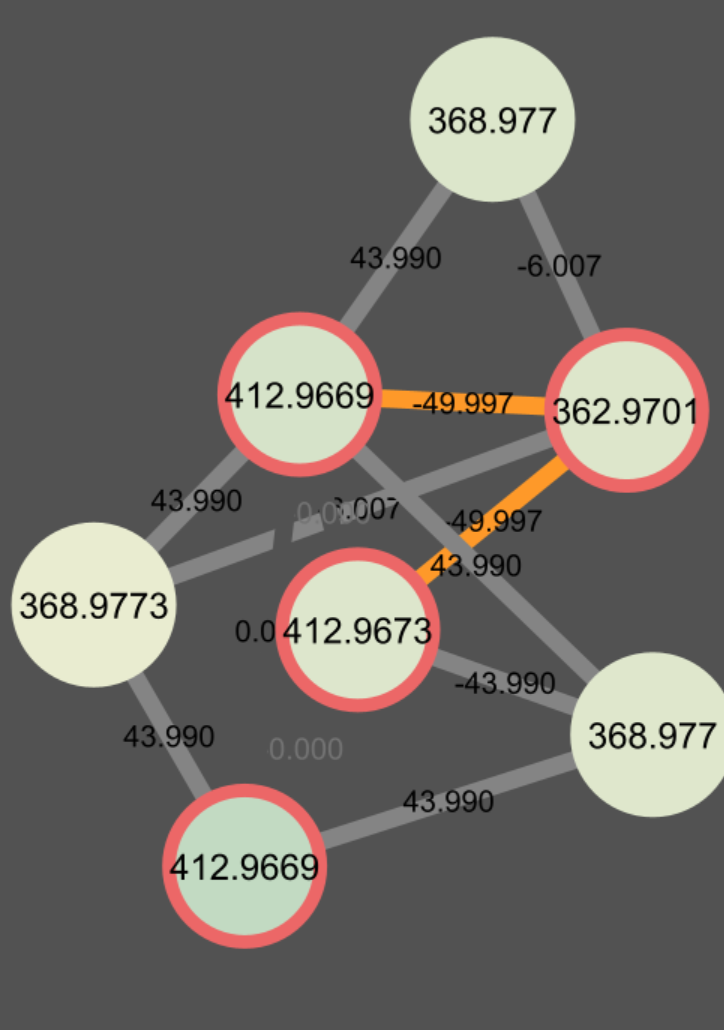


**Figure S7.** Molecular network of polyfluorinated acids. 362.9701 is 7-tridecafluoroenanthic acid; 412.9669 and 412.9673 are 8-pentadecafluorooctanoic acid. 368.977 and 368.9773 remained unidentified. The orange line denotes a m/z difference corresponding to the loss or gain of a CF_2_ unit.


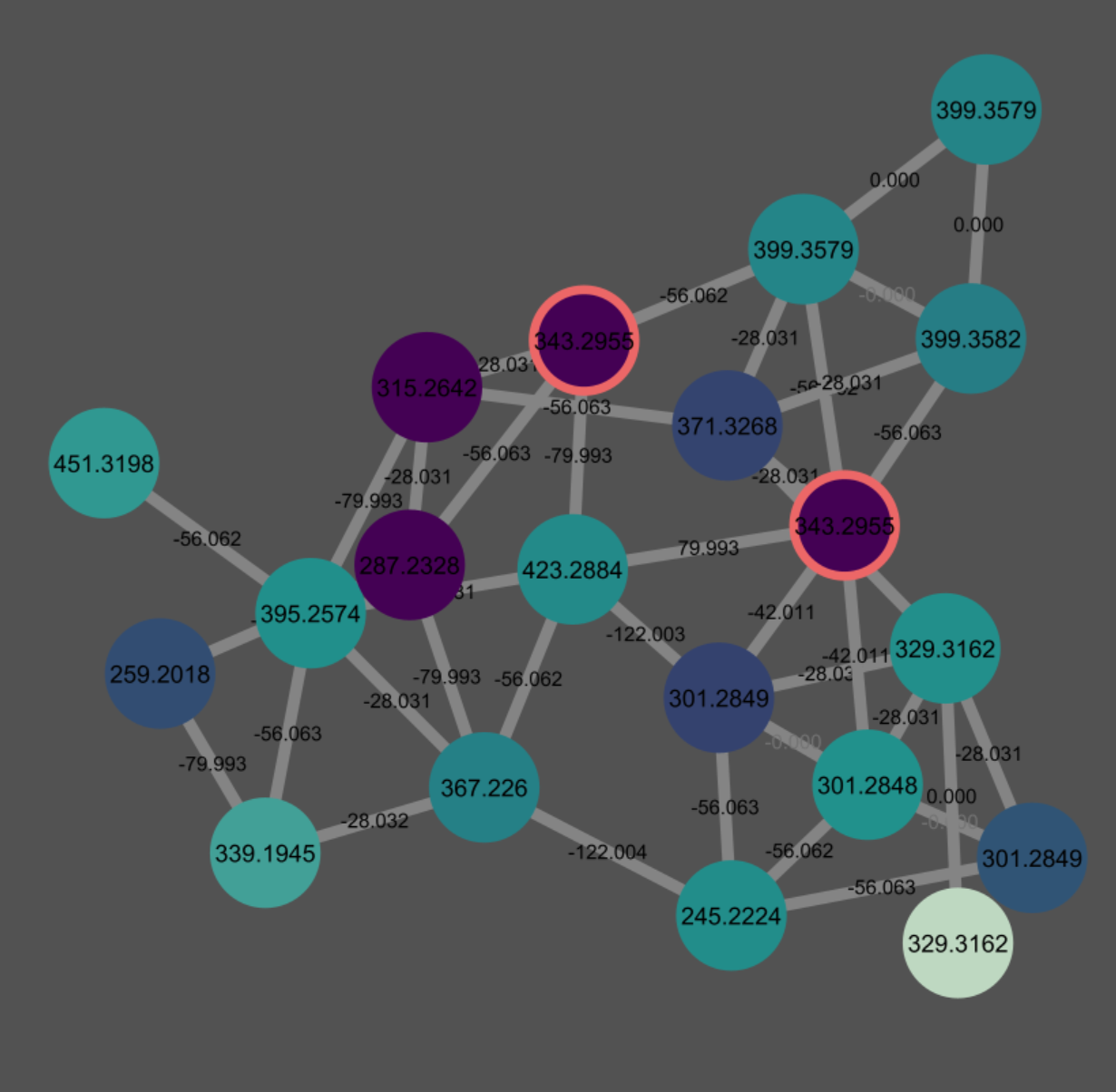


**Figure S8.** Molecular network of alkylamidopropylbetaines. 245.2224 is Pentamidopropyl betaine; 259.2018 is Hexamidopropyl betaine; 267.2328 is Caprylamidopropyl betaine; 301.2849 is Nonamidopropyl betaine; 315.2642 is Decamidopropyl betaine; 329.3162 is Undecamidopropyl betaine; 343,2955 is Lauramidopropyl betaine; 371.3268 is Myristamidopropyl betaine; 399.3579 is Hexadecamidopropyl betaine.


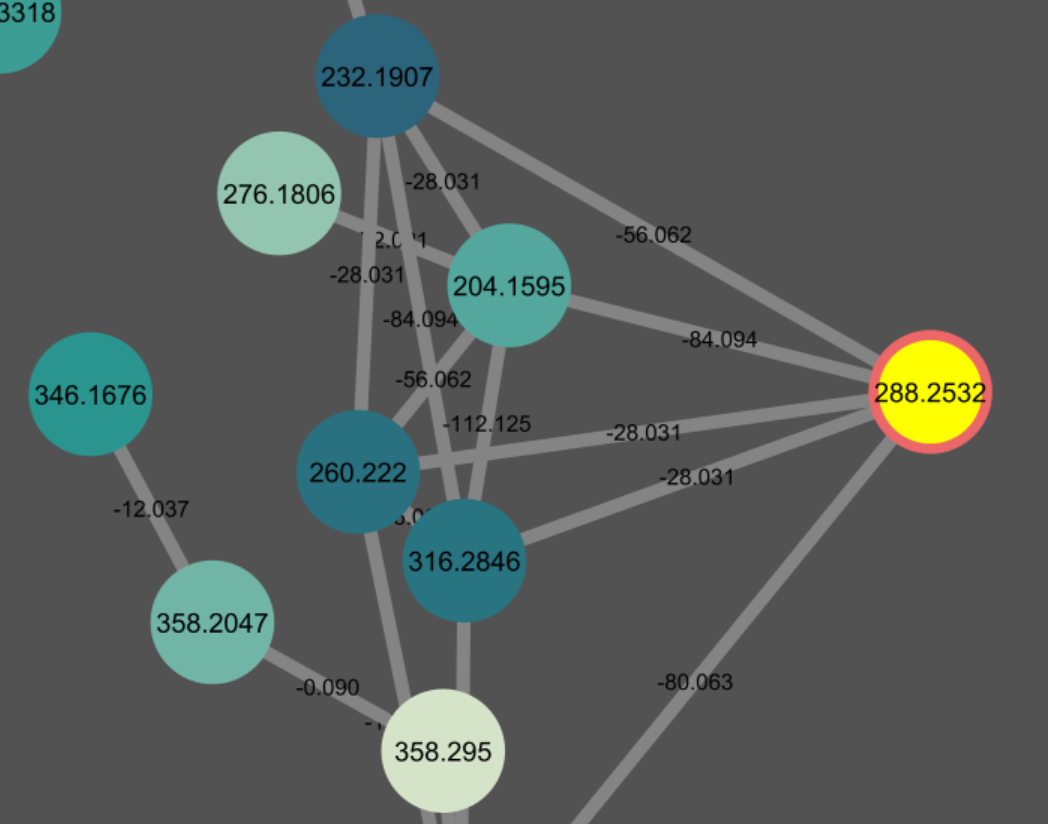


**Figure S9.** Molecular network of alkyldiethanolamides. 204.1595 is Hexyl diethanolamide; 232.1907 is Octyl diethanolamide; 260.222 is Decyl diethanolamide; 288.2532 is Lauryl diethanolamide; 316.2846 is Tetradecyl diethanolamide; 358.295 is Heptadecyl diethanolamide. 358.2047 is a coeluting isobar of 358.295.


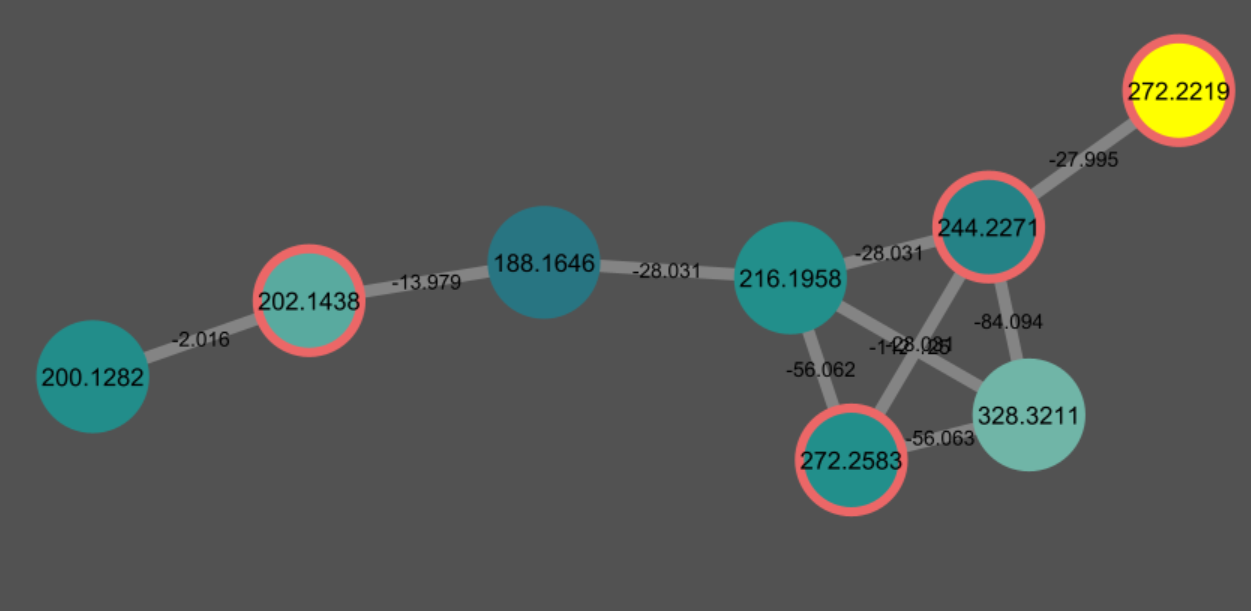


**Figure S10.** Molecular network of alkylethanolamides. 188.1646 is Octyl ethanolamide; 216.1958 is Decyl ethanolamide; 244.2271 is Dodecyl ethanolamide; 272.2583 is Myristoyl Ethanolamide; 328.3211 is Stearoyl ethanolamide.


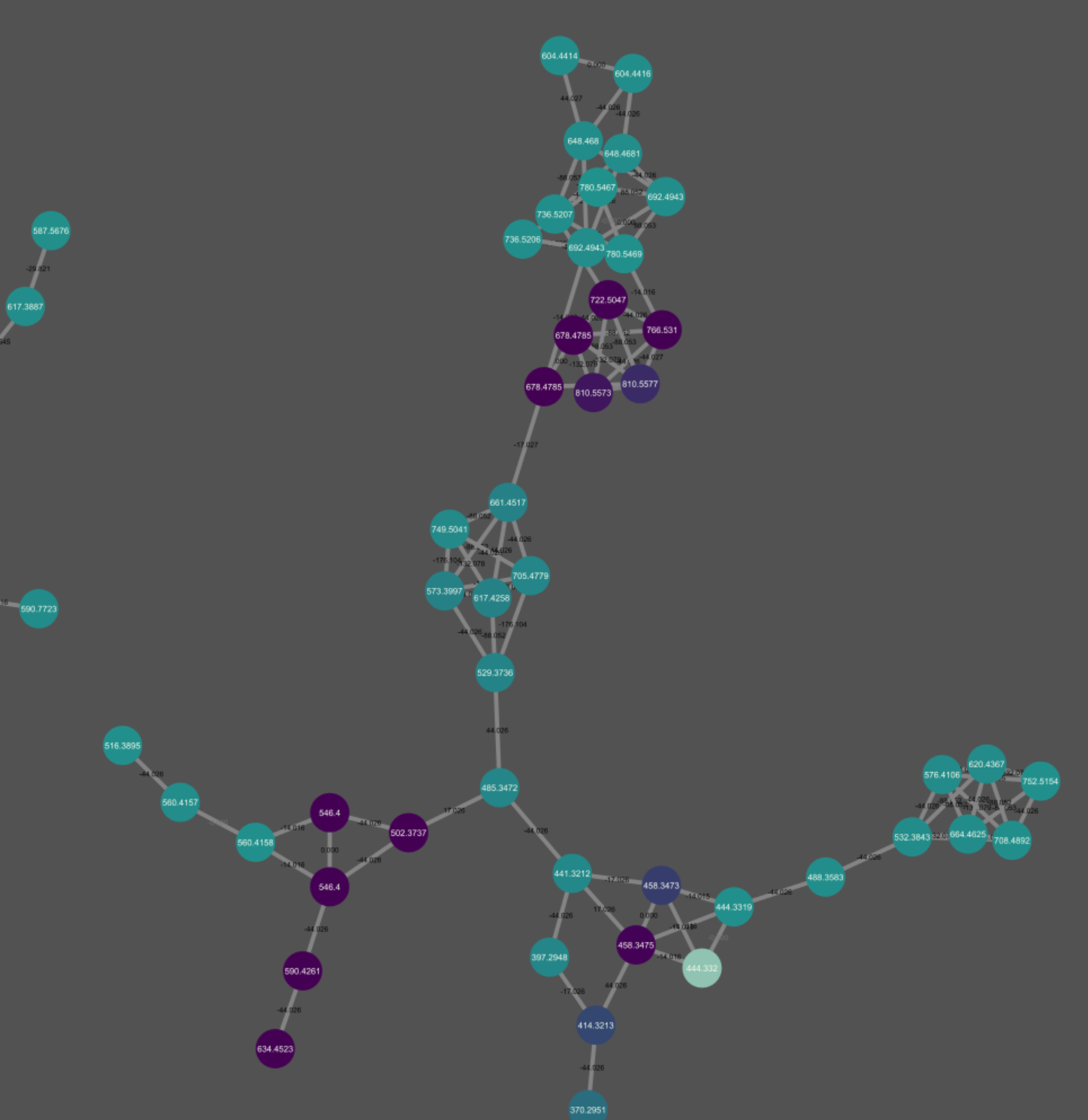


**Figure S11.** Molecular network of nonylphenol ethoxylates congeners. 370.2951 is NPEO-02, 414.3213 is NPEO-03, 458.3473 is NPEO-04, 502.3737 is NPEO-05, 546.4000 is NPEO-06, 590.4261 is NPEO-07, 634.4523 is NPEO-08, 678.4785 is NPEO-09, 722.5047 is NPEO-10, 766.531 is NPEO-11, 810.5577 is NPEO-12.


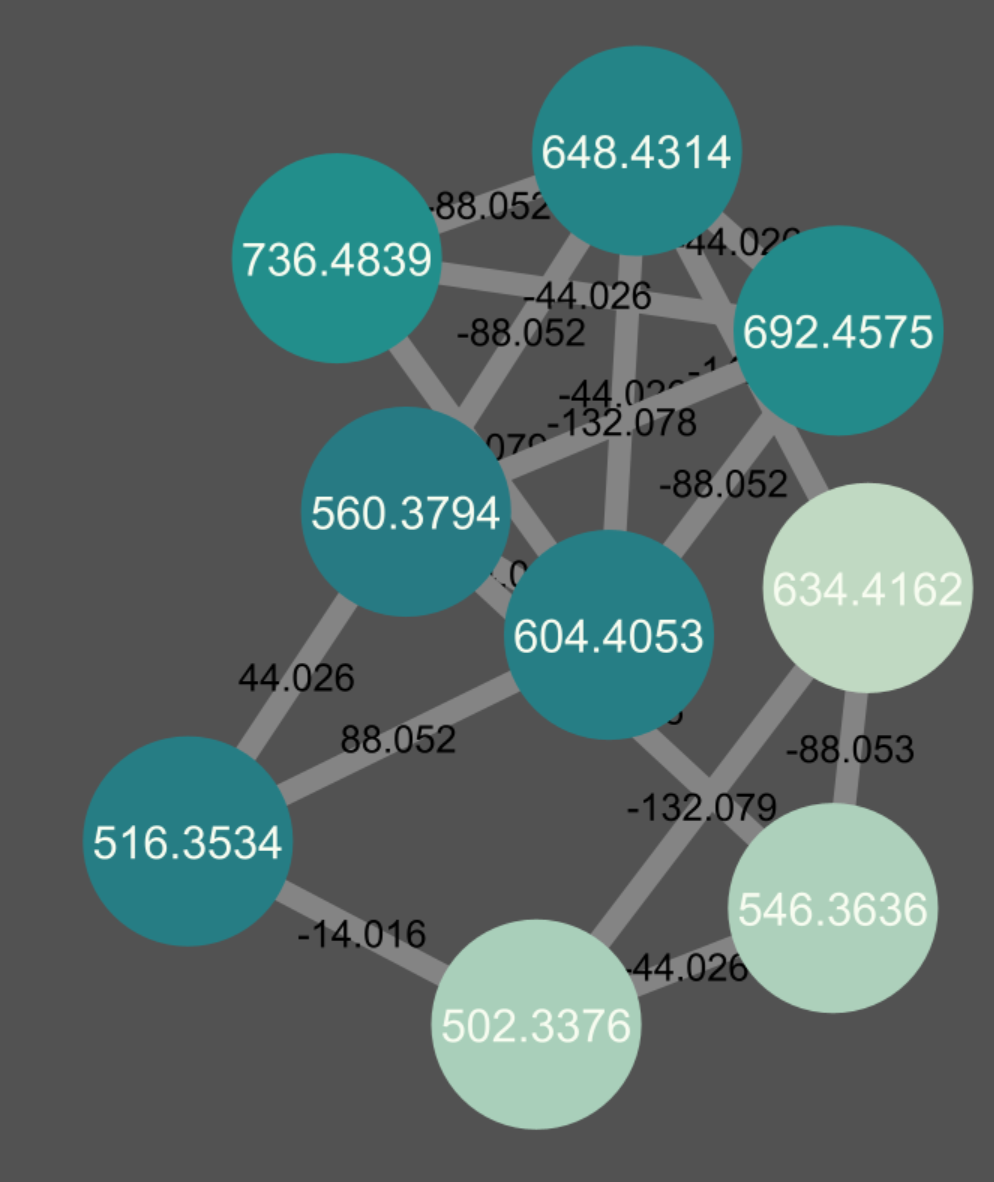


**Figure S12.** Molecular network of octylphenol ethoxyacids (NPEOAs) and nonylphenol ethoxyacids (OPEOAs). 516.3534 is NPEOA-05, 560.3794 is NPEOA-06, 604.4053 is NPEOA-07, 648.4314 is NPEOA-08, 692.4575 is NPEOA-09, 736.4839 is NPEOA-10, 502.3376 is OPEOA-05, 546.3636 is OPEOA-06, 634.4162 is OPEOA-07.


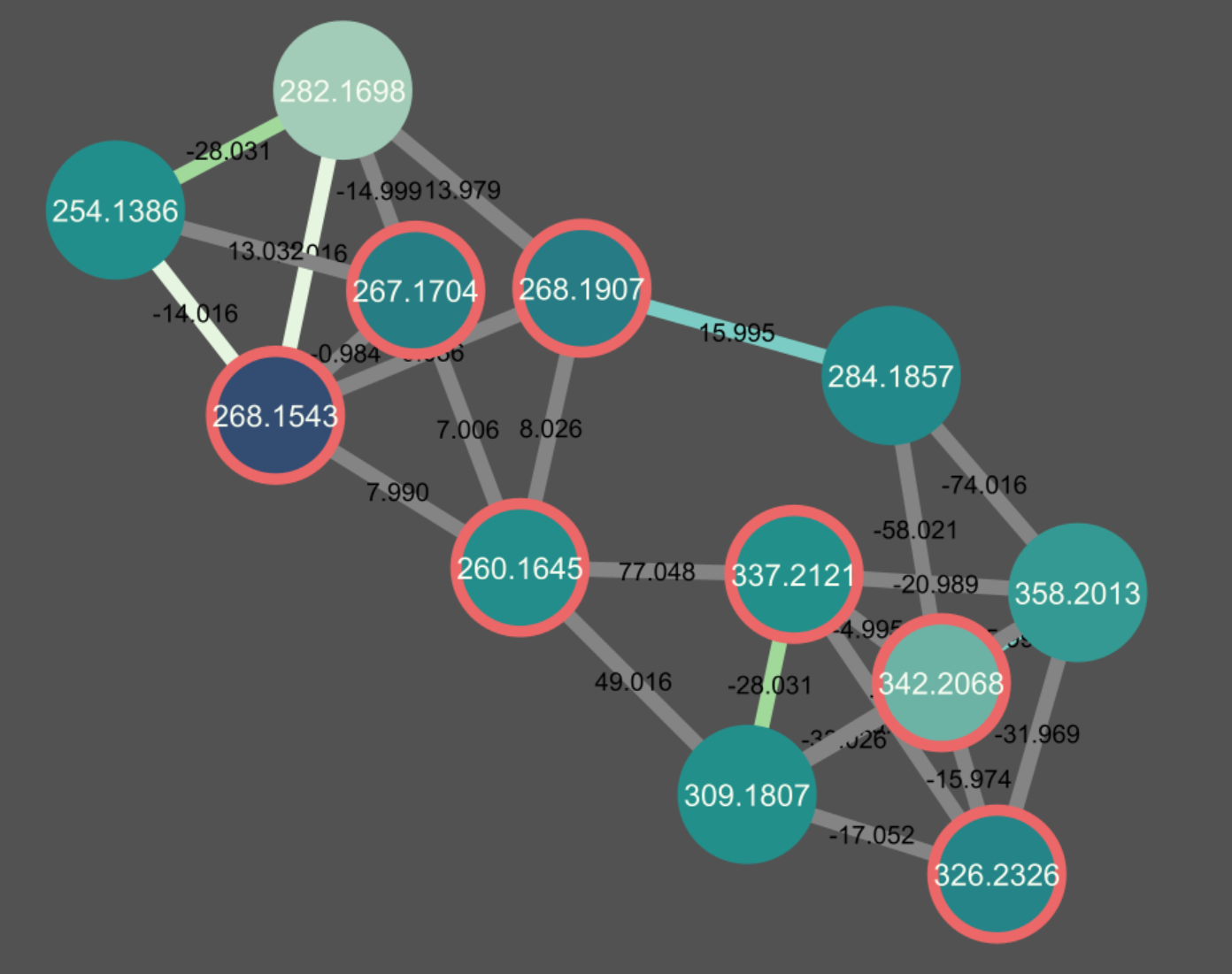


**Figure S13.** Molecular network of beta-blockers and TPs. 254.1386 is Desmethyl Metoprolol acid; 260.1645 is Propranolol; 267.1704 is atenolol; 268.1543 is Metoprolol acid; 268.1907 is Metoprolol; 282.1698 is Metoprolol Acid methyl Ester; 284.1857 is Hydroxymetoprolol; 309.1807 is Desethylacebutolol; 326.2326 is Bisoprolol; 337.2121 is acebutolol; 342.2068 is Propafenone; and 358.2013 is Hydroxypropafenone.


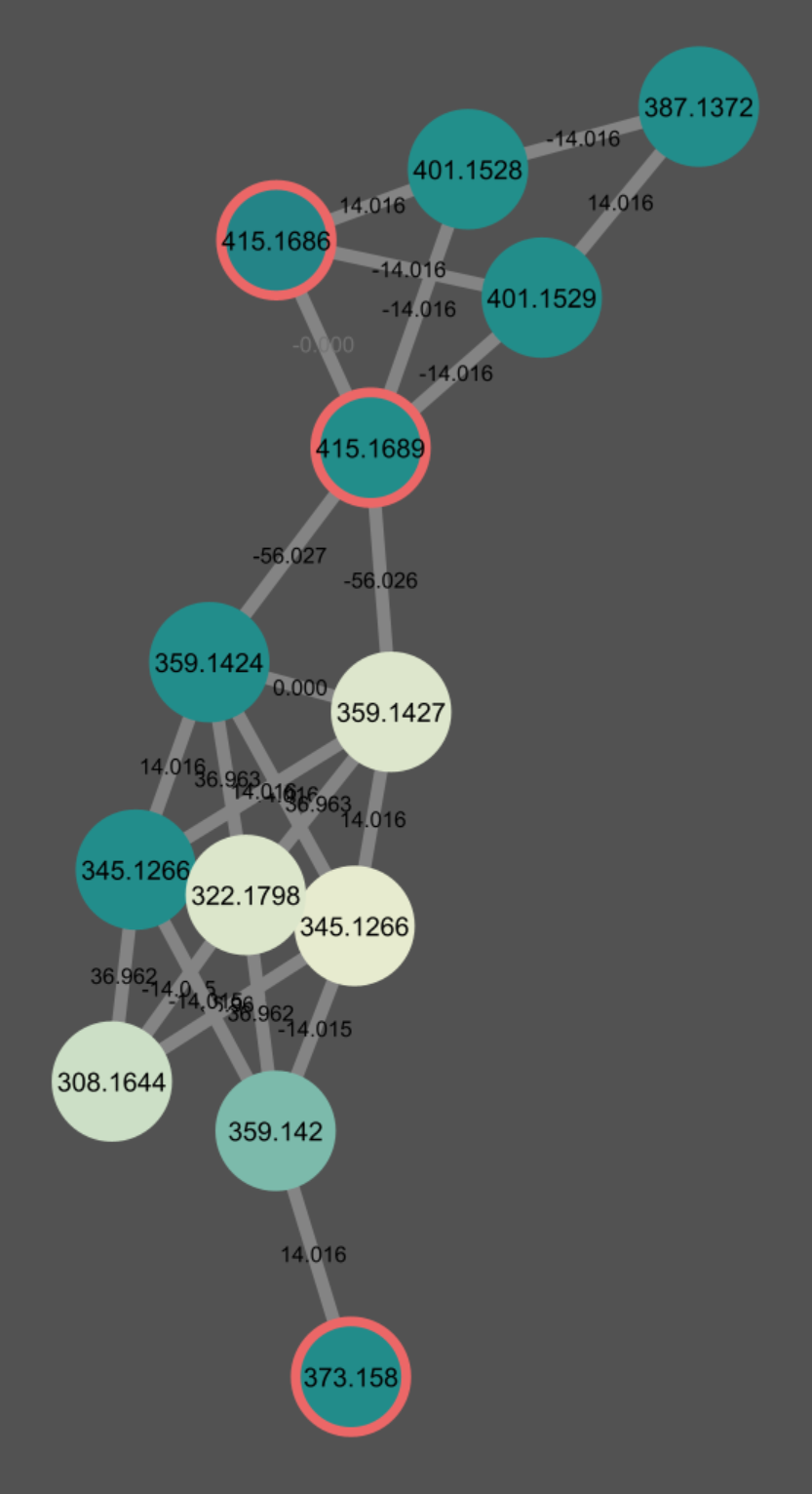


**Figure S14.** Molecular network of the calcium channel blocker diltiazem and its TPs. 415.1686 and 415.1689 are diltiazem; 401.1528, 401.1529 are demethyldiltiazem; 387.1372 is didemethyldiltiazem; 373.158 is desacetyldiltiazem; 359.1424, 359.1427, 359.142 are demethyldesacetyldiltiazem and 345.1266 is didemethyldeacetyldiltiazem. 322.1798 and 308.1644 remained unidentified.


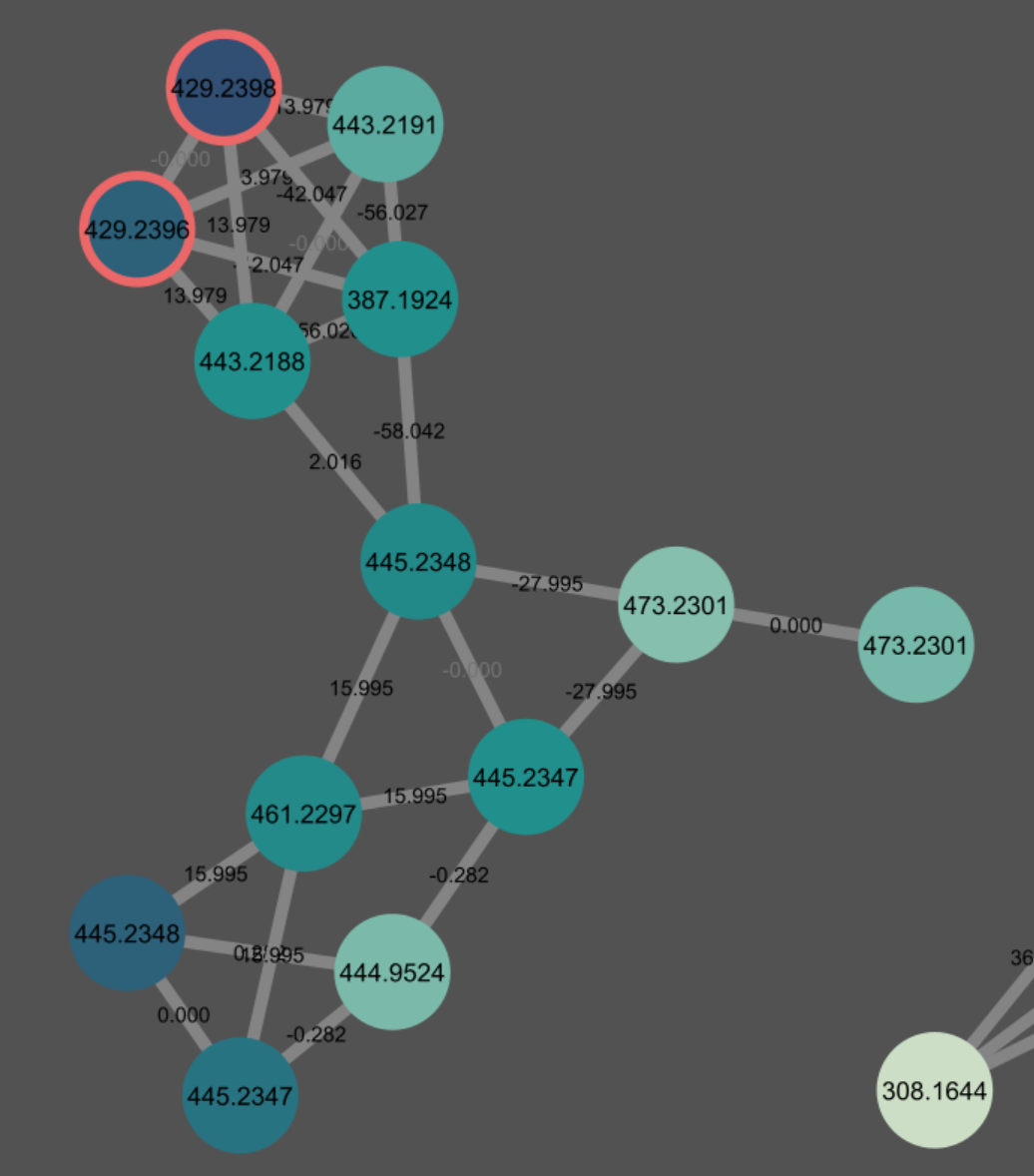


**Figure S15.** Molecular network of the angiotensin II receptor antagonist irbesartan and 5 of its TPs. 429.2396 and 429.2398 are irbesartan; 473.2301 is Irbesartan_TP473; 461.2297 is Dihydroxyirbesartan; 445.2348 and 445.2347 are Hydroxyirbesartan; 443.2188 and 443.2191 are irbesartan aldehyde and 387.1924 is despropylirbesartan.


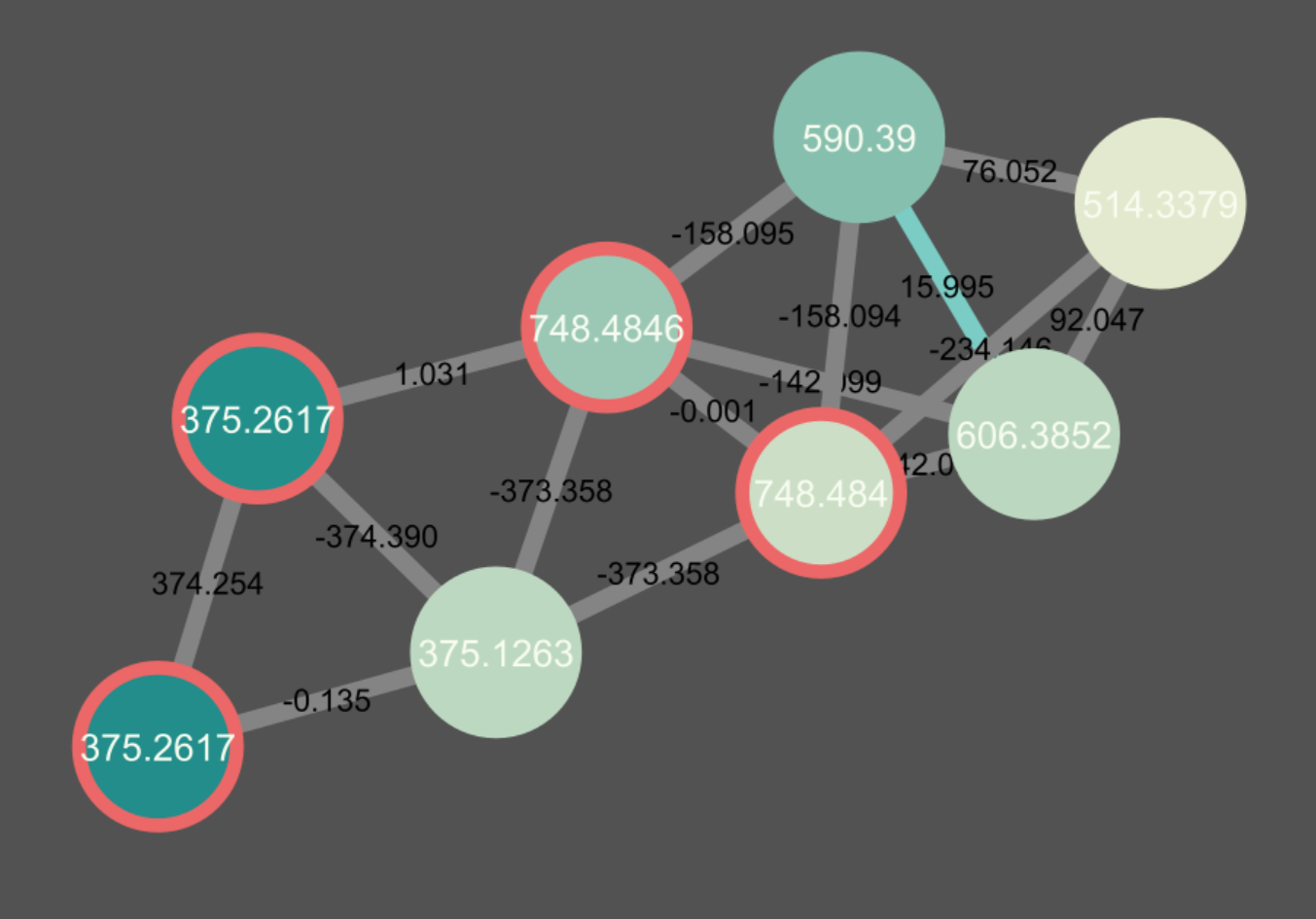


**Figure S16.** Molecular network of azithromycin, clarithromycin and related compounds. 375.2617 is doubly charged azithromycin while 748.484 and 748.4846 are also clarithromycin with a single charge.


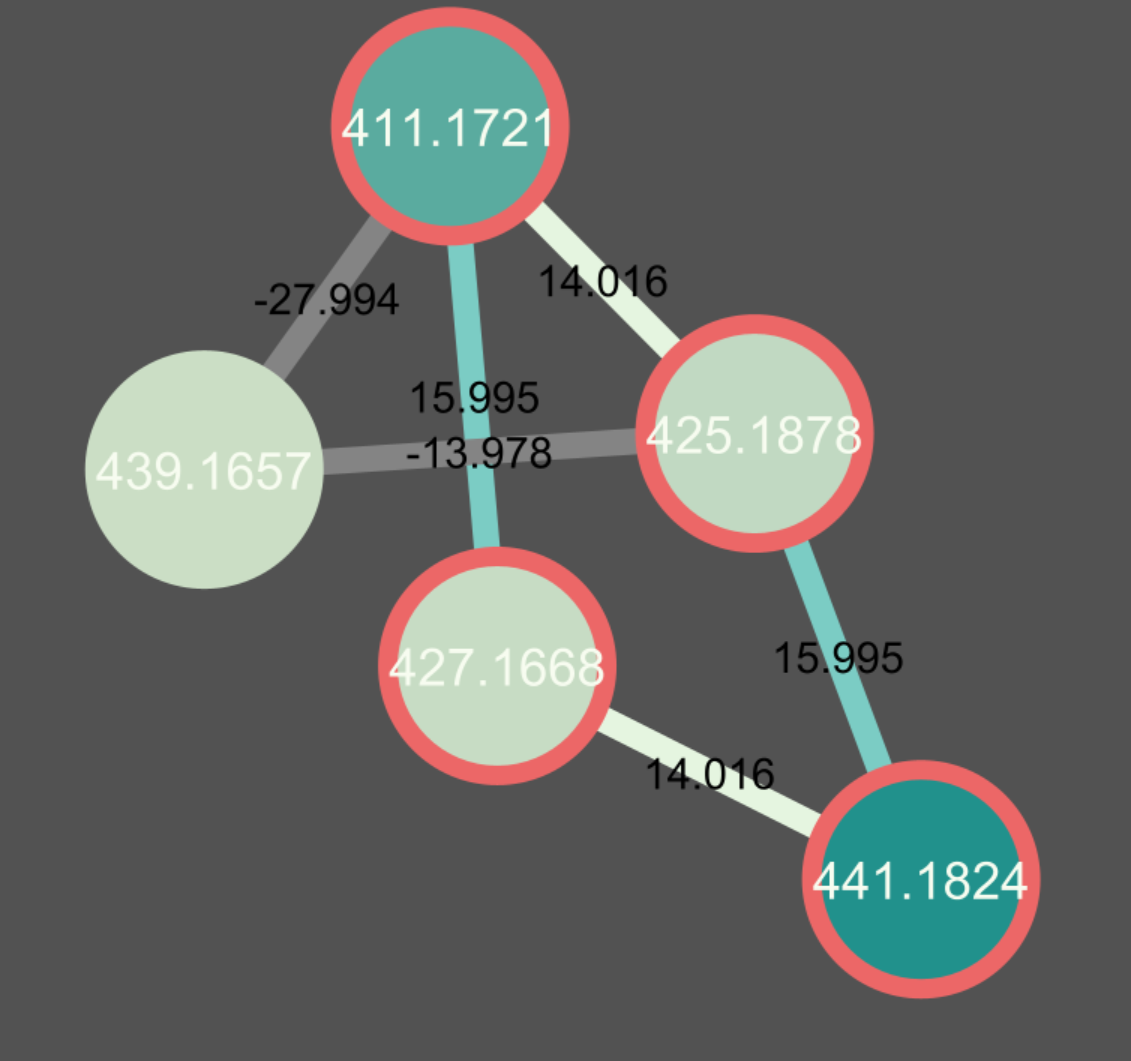


**Figure S17.** Molecular network of clindamycin (425.1878) along with its transformation products clindamycin sulfoxide (441.1824), N-desmethylclindamycin (411.1721), N-desmethylclindamycin sulfoxide (427.1668) and clindamycin_TP439 (439.1657)


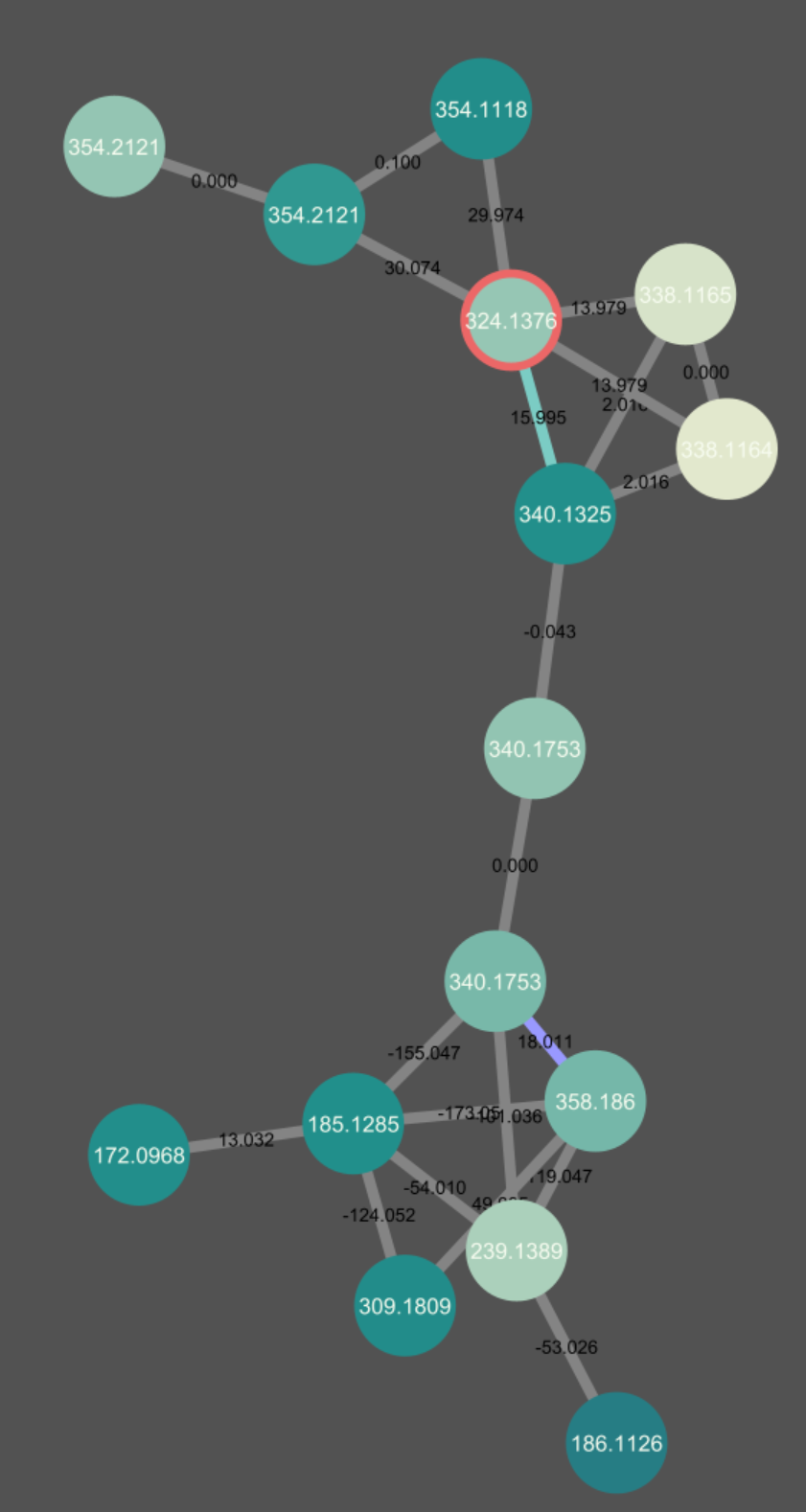


**Figure S18.** Molecular network of gliclazide (324.1376) and its transformation products hydroxygliclazide (340.1325), gliclazide aldehyde (338.1164 and 338.1165) and carboxygliclazide (354.1118).


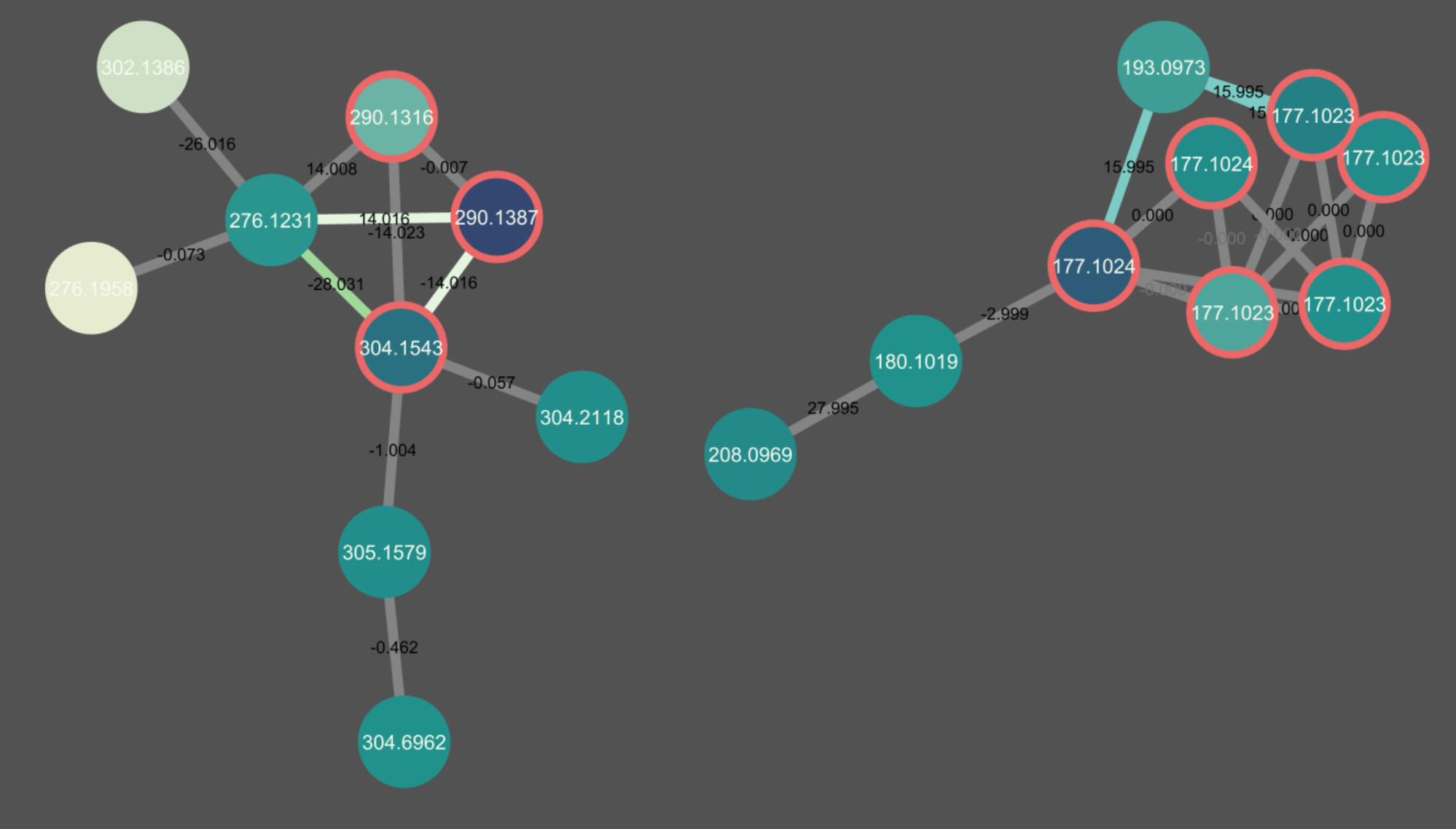


**Figure S19.** Molecular network of cocaine (304.1543), its main metabolite benzoylecgonine (290.1387) and transformation product norbenzoylecgonine (276.1231).

1. Overdahl KE, Sutton R, Sun J, DeStefano NJ, Getzinger GJ, Ferguson PL (2021) Assessment of emerging polar organic pollutants linked to contaminant pathways within an urban estuary using non-targeted analysis. Environ Sci Process Impacts 23:429–445. https://doi.org/10.1039/D0EM00463D

2. Qian Y, Wang X, Wu G, Wang L, Geng J, Yu N, Wei S (2021) Screening priority indicator pollutants in full-scale wastewater treatment plants by non-target analysis. J Hazard Mater 414:125490. https://doi.org/10.1016/j.jhazmat.2021.125490

3. Campos-Mañas MC, Plaza-Bolaños P, Martínez-Piernas AB, Sánchez-Pérez JA, Agüera A (2019) Determination of pesticide levels in wastewater from an agro-food industry: Target, suspect and transformation product analysis. Chemosphere 232:152–163. https://doi.org/10.1016/j.chemosphere.2019.05.147

4. Čelić M, Jaén-Gil A, Briceño-Guevara S, Rodríguez-Mozaz S, Gros M, Petrović M (2021) Extended suspect screening to identify contaminants of emerging concern in riverine and coastal ecosystems and assessment of environmental risks. J Hazard Mater 404:124102. https://doi.org/10.1016/j.jhazmat.2020.124102

5. Choi Y, Lee J-H, Kim K, Mun H, Park N, Jeon J (2021) Identification, quantification, and prioritization of new emerging pollutants in domestic and industrial effluents, Korea: Application of LC-HRMS based suspect and non-target screening. J Hazard Mater 402:123706. https://doi.org/10.1016/j.jhazmat.2020.123706

6. Perkons I, Rusko J, Zacs D, Bartkevics V (2021) Rapid determination of pharmaceuticals in wastewater by direct infusion HRMS using target and suspect screening analysis. Sci Total Environ 755:142688. https://doi.org/10.1016/j.scitotenv.2020.142688

7. Challis JK, Almirall XO, Helm PA, Wong CS (2020) Performance of the organic-diffusive gradients in thin-films passive sampler for measurement of target and suspect wastewater contaminants. Environ Pollut 261:114092. https://doi.org/10.1016/j.envpol.2020.114092

8. Wielens Becker R, Ibáñez M, Cuervo Lumbaque E, Wilde ML, Flores da Rosa T, Hernández F, Sirtori C (2020) Investigation of pharmaceuticals and their metabolites in Brazilian hospital wastewater by LC-QTOF MS screening combined with a preliminary exposure and in silico risk assessment. Sci Total Environ 699:134218. https://doi.org/10.1016/j.scitotenv.2019.134218

9. Ccanccapa-Cartagena A, Pico Y, Ortiz X, Reiner EJ (2019) Suspect, non-target and target screening of emerging pollutants using data independent acquisition: Assessment of a Mediterranean River basin. Sci Total Environ 687:355–368. https://doi.org/10.1016/j.scitotenv.2019.06.057

10. Zwart N, Jonker W, Broek R ten, de Boer J, Somsen G, Kool J, Hamers T, Houtman CJ, Lamoree MH (2020) Identification of mutagenic and endocrine disrupting compounds in surface water and wastewater treatment plant effluents using high-resolution effect-directed analysis. Water Res 168:115204. https://doi.org/10.1016/j.watres.2019.115204

11. Gago-Ferrero P, Bletsou AA, Damalas DE, Aalizadeh R, Alygizakis NA, Singer HP, Hollender J, Thomaidis NS (2020) Wide-scope target screening of >2000 emerging contaminants in wastewater samples with UPLC-Q-ToF-HRMS/MS and smart evaluation of its performance through the validation of 195 selected representative analytes. J Hazard Mater 387:121712. https://doi.org/10.1016/j.jhazmat.2019.121712

12. Liu W, Yao H, Xu W, Liu G, Wang X, Tu Y, Shi P, Yu N, Li A, Wei S (2020) Suspect screening and risk assessment of pollutants in the wastewater from a chemical industry park in China. Environ Pollut 263:114493. https://doi.org/10.1016/j.envpol.2020.114493

13. Stincone P, Pakkir Shah AK, Schmid R, Graves L, P. Lambidis S, Torres R, Xia S-N, Minda V, Aron A, Wang M, Hughes CC, Petras D (2023) Evaluation of Data Dependent MS/MS Acquisition Parameters for Non-targeted Metabolomics and Molecular Networking of Environmental Samples - Focus on the Q Exactive Platform. Chemistry

14. Redestig H, Fukushima A, Stenlund H, Moritz T, Arita M, Saito K, Kusano M (2009) Compensation for Systematic Cross-Contribution Improves Normalization of Mass Spectrometry Based Metabolomics Data. Anal Chem 81:7974–7980. https://doi.org/10.1021/ac901143w

15. SUGIHARA J, SUGAWARA Y, ANDO H, HARIGAYA S, ETOH A, KOHNO K (1984) Studies on the metabolism of diltiazem in man. J Pharmacobiodyn 7:24–32

16. Yeung PKF, Prescott C, Haddad C, Montague TJ, McGregor C, Quilliam MA, Xei M, Li R, Farmer P, Klassen GA (1993) Pharmacokinetics and metabolism of diltiazem in healthy males and females following a single oral dose. Eur J Drug Metab Pharmacokinet 18:199–206. https://doi.org/10.1007/BF03188796

17. Molden E, Åsberg A, Christensen H (2002) Desacetyl-diltiazem displays severalfold higher affinity to CYP2D6 compared with CYP3A4. Drug Metab Dispos 30:1–3

18. Eysseric E, Gagnon C, Segura PA (2022) Identifying congeners and transformation products of organic contaminants within complex chemical mixtures in impacted surface waters with a top-down non-targeted screening workflow. Sci Total Environ 822:153540. https://doi.org/10.1016/j.scitotenv.2022.153540

19. Eysseric E, Beaudry F, Gagnon C, Segura PA (2021) Non-targeted screening of trace organic contaminants in surface waters by a multi-tool approach based on combinatorial analysis of tandem mass spectra and open access databases. Talanta 230:122293. https://doi.org/10.1016/j.talanta.2021.122293

20. Oberleitner D, Schmid R, Schulz W, Bergmann A, Achten C (2021) Feature-based molecular networking for identification of organic micropollutants including metabolites by non-target analysis applied to riverbank filtration. Anal Bioanal Chem 413:5291–5300. https://doi.org/10.1007/s00216-021-03500-7

21. Boix C, Ibáñez M, Sancho JV, Parsons JR, Voogt P de, Hernández F (2016) Biotransformation of pharmaceuticals in surface water and during waste water treatment: Identification and occurrence of transformation products. J Hazard Mater 302:175–187. https://doi.org/10.1016/j.jhazmat.2015.09.053

22. Eysseric E, Gagnon C, Segura PA (2022) Uncovering transformation products of four organic contaminants of concern by photodegradation experiments and analysis of real samples from a local river. Chemosphere 293:133408. https://doi.org/10.1016/j.chemosphere.2021.133408

23. Konstas P– S, Hela D, Giannakas A, Triantafyllos A, Konstantinou I (2019) Photocatalytic degradation of organophosphate flame retardant TBEP: kinetics and identification of transformation products by orbitrap mass spectrometry. Int J Environ Anal Chem 99:297–309. https://doi.org/10.1080/03067319.2019.1593399
